# Supplementary material for: Cladolosides of Groups S and T: Triterpene Glycosides from the Sea Cucumber Cladolabes schmeltzii with Unique Sulfation; Human Breast Cancer Cytotoxicity and QSAR
Source: Mar Drugs. 2025 Jun 25;23(7):265. doi: 10.3390/md23070265 (PMC12299463; doi:10.3390/md23070265)
Supplement: Supplementary file 1 [file marinedrugs-23-00265-s001.zip › Supporting.pdf]

Supplementary data content page

Title: Cladolosides S, S_1, T, and T_1 – Triterpene Glycosides from the Sea Cucumber *Cladolabes schmeltzii*: Unique Sulfate Group Position, Cytotoxicity against Human Breast Cancer Cell Lines; Quantitative Structure–Activity Relationships

Authors: Alexandra S. Silchenko [1,*], Elena A. Zelepuga [1], Ekaterina A. Chingizova [1], Ekaterina S. Menchinskaya [1], Kseniya M. Tabakmakher [1], Anatoly I. Kalinovsky [1], Sergey A. Avilov [1], Roman S. Popov [1], Pavel S. Dmitrenok [1], and Vladimir I. Kalinin [1]

Address: [1]*G.B. Elyakov Pacific Institute of Bioorganic Chemistry, Far Eastern Branch of Russian Academy of Sciences, Pr. 100-let Vladivostoka 159, 690022 Vladivostok, Russia*

Correspondence: silchenko_als@piboc.dvo.ru; Tel.: +7-423-231-1168

Content:

S1. Methodology of acid hydrolysis and determination of absolute configurations of monosaccharides.

Figure S1. The ^{13}C NMR (176.04 MHz) spectrum of cladoloside S (**1**) in C_5D_5N/D_2O (4/1)

Figure S2. The ^{1}H NMR (700.13 MHz) spectrum of cladoloside S (**1**) in C_5D_5N/D_2O (4/1)

Figure S3. The COSY (700.13 MHz) spectrum of cladoloside S (**1**) in C_5D_5N/D_2O (4/1)

Figure S4. The HSQC (700.13 MHz) spectrum of cladoloside S (**1**) in C_5D_5N/D_2O (4/1)

Figure S5. The ROESY (700.13 MHz) spectrum of cladoloside S (**1**) in C_5D_5N/D_2O (4/1)

Figure S6. The HMBC (700.13 MHz) spectrum of cladoloside S (**1**) in C_5D_5N/D_2O (4/1)

Figure S7. 1 D TOCSY (700.13 MHz) spectra of Xyl1, Qui2, Xyl3, MeGlc4, Xyl5 of cladoloside S (**1**) in C_5D_5N/D_2O (4/1)

Figure S8. HR-ESI-MS and ESI-MS/MS spectra of cladoloside S (**1**)

Figure S9. The ^{13}C NMR (176.04 MHz) spectrum of cladoloside S_1 (**2**) in C_5D_5N/D_2O (4/1)

Figure S10. The ^{1}H NMR (700.13 MHz) spectrum of cladoloside S_1 (**2**) in C_5D_5N/D_2O (4/1)

Figure S11. The COSY (700.13 MHz) spectrum of cladoloside S_1 (**2**) in C_5D_5N/D_2O (4/1)

Figure S12. The HSQC (700.13 MHz) spectrum of cladoloside S_1 (**2**) in C_5D_5N/D_2O (4/1)

Figure S13. The ROESY (700.13 MHz) spectrum of cladoloside S_1 (**2**) in C_5D_5N/D_2O (4/1)

Figure S14. The HMBC (700.13 MHz) spectrum of cladoloside S_1 (**2**) in C_5D_5N/D_2O (4/1)

Figure S15. 1 D TOCSY (700.13 MHz) spectra of Xyl1, Qui2, Xyl3, MeGlc4, Xyl5 of cladoloside S_1 (**2**) in C_5D_5N/D_2O (4/1)

Figure S16. HR-ESI-MS and ESI-MS/MS spectra of cladoloside S_1 (**2**)

Table S1. One- and two-dimensional NMR data of oligosaccharide moiety of cladoloside S_1 (**2**)

Figure S17. The ^{13}C NMR (176.04 MHz) spectrum of cladoloside T (**3**) in C_5D_5N/D_2O (4/1)

Figure S18. The ^{1}H NMR (700.13 MHz) spectrum of cladoloside T (**3**) in C_5D_5N/D_2O (4/1)

Figure S19. The COSY (700.13 MHz) spectrum of cladoloside T (**3**) in C_5D_5N/D_2O (4/1)

Figure S20. The HSQC (700.13 MHz) spectrum of cladoloside T (**3**) in C_5D_5N/D_2O (4/1)

Figure S21. The ROESY (700.13 MHz) spectrum of cladoloside T (**3**) in C_5D_5N/D_2O (4/1)

Figure S22. The HMBC (700.13 MHz) spectrum of cladoloside T (**3**) in C_5D_5N/D_2O (4/1)

Figure S23. 1 D TOCSY (700.13 MHz) spectra of Xyl1, Qui2, Xyl3, Glc4, Glc5, MeGlc6 of cladoloside T (**3**) in C_5D_5N/D_2O (4/1)

Table S2. One- and two-dimensional NMR data of the aglycone moiety of cladoloside T (**3**)

Figure S24. HR-ESI-MS and ESI-MS/MS spectra of cladoloside T (**3**)

Figure S25. The ^{13}C NMR (176.04 MHz) spectrum of cladoloside T_1 (**4**) in C_5D_5N/D_2O (4/1)

Figure S26. The ^{1}H NMR (700.13 MHz) spectrum of cladoloside T_1 (**4**) in C_5D_5N/D_2O (4/1)

Figure S27. The COSY (700.13 MHz) spectrum of cladoloside T_1 (**4**) in C_5D_5N/D_2O (4/1)

Figure S28. The HSQC (700.13 MHz) spectrum of cladoloside T_1 (**4**) in C_5D_5N/D_2O (4/1)

Figure S29. The ROESY (700.13 MHz) spectrum of cladoloside T_1 (**4**) in C_5D_5N/D_2O (4/1)

Figure S30. The HMBC (700.13 MHz) spectrum of cladoloside T_1 (**4**) in C_5D_5N/D_2O (4/1)

Figure S31. 1 D TOCSY (700.13 MHz) spectra of Xyl1, Qui2, Xyl3, Glc4, Glc5, MeGlc6 of cladoloside T_1 (**4**) in C_5D_5N/D_2O (4/1)

Figure S32. HR-ESI-MS and ESI-MS/MS spectra of cladoloside T_1 (**4**)

Table S3. One- and two-dimensional NMR data of the aglycone moiety of cladoloside T_1 (**4**)

Table S4. One- and two-dimensional NMR data of oligosaccharide moiety of cladoloside T_1 (**4**)

Figure S33. The structure of glycosides, isolated earlier from the sea cucumber *Cladolabes schmeltzii*

Figure S34. The PLS QSAR model correlation plot

Figure S35. The correlation matrix of descriptors impacts (43 variables) to the hemolytic (pED50, μM) and cytotoxic activities (pIC50, μM) against MCF-10A, MCF-7 and MDA-MB-231 cells of 26 glycosides constructed with the QuaSAR-Descriptor tool of MOE 2020.0901 CCG software.

S1. Methodology of acid hydrolysis and determination of absolute configurations of monosaccharides.

The acid hydrolysis of the glycosidic fraction, including the cladolosides of the groups S and T was conducted in a solution of 0.2 M trifluoroacetic acid (TFA) (300 ml) in a sealed vial on a water bath at 100 °C for 1 h. The H_2O layer was extracted with $CHCl_3$ (3×500 ml) and concentrated in vacuo. One drop of concentrated TFA and 200 ml of R-(−)-2-octanol (Aldrich) was added to the dry residue of the sugars. The ampoule was sealed and heated on a glycerol bath at 130 °C for 6 h. The obtained derivatives were evaporated in vacuo and treated with a mixture of pyridine/acetic anhydride (1:1, 600 ml) for 24 h at room temperature. The acetylated (−)-2-octylglycosides were analyzed by GLC using corresponding authentic samples: D-xylose, D-quinovose, D-glucose, 3-O-methyl-D-glucose treated by the same procedure. The following peaks were detected: D-xylose (retention times 24.57, 24.76 and 25.20 min), D-quinovose (retention times 24.00, 24.25, 24.69, 24.91 min), D-glucose (retention times 28.23, 28.87, 29.08, 29.34 min) and 3-O-methyl-D-glucose (28.23, 28.55, 28.85, 29.58 min). Retention times of the authentic samples were as follows: D-xylose (24.57, 24.76 and 25.1 min), D-quinovose (23.99, 24.24, 24.67 and 24.89 min), D-glucose (28.22, 28.86, 29.06 and 29.32 min) and 3-O-methyl-D-glucose (28.23, 28.56, 28.84 and 29.59 min).

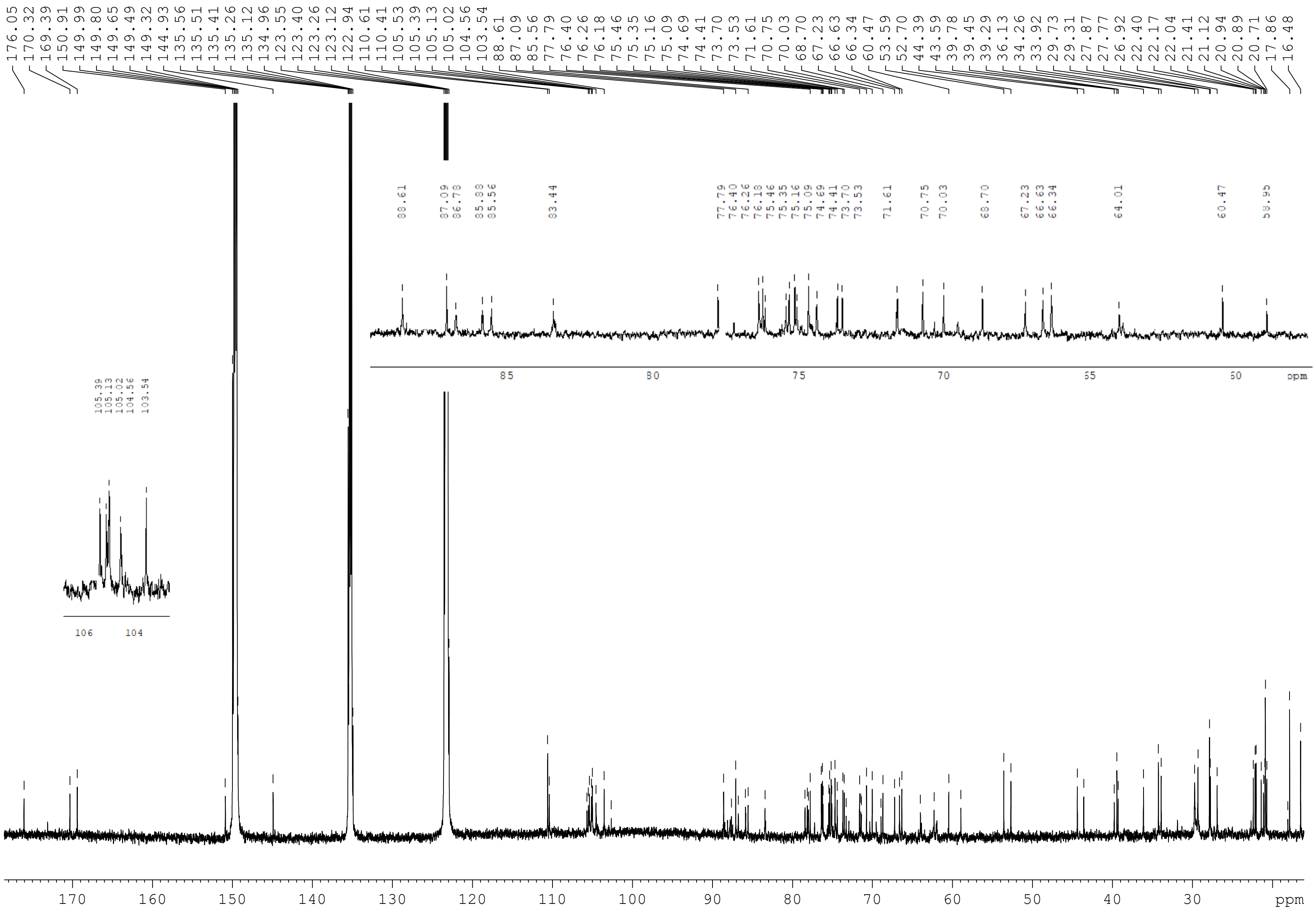

Figure S1. The ^{13}C NMR (176.04 MHz) spectrum of cladoloside S (**1**) in C_5D_5N/D_2O (4/1)

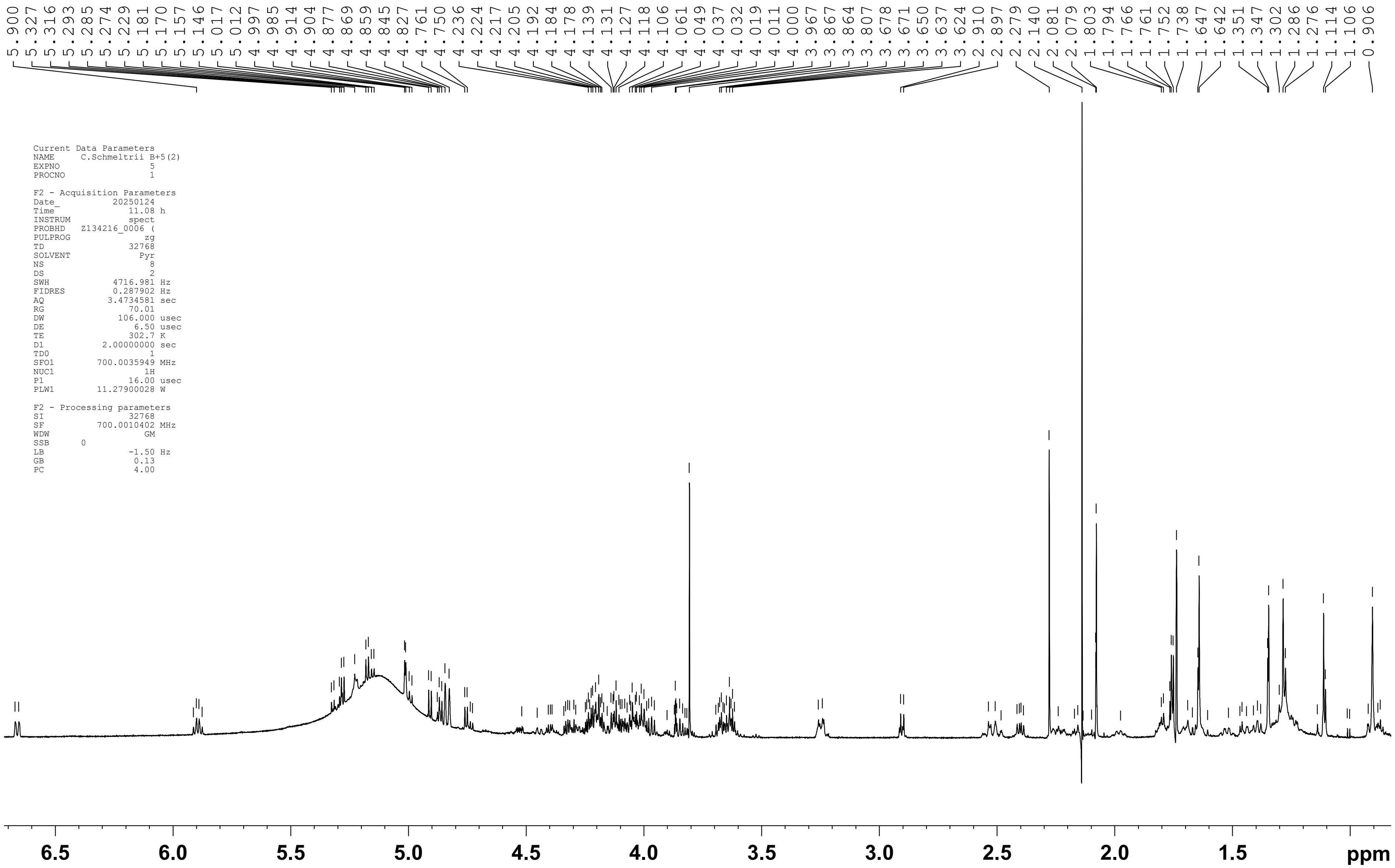

Figure S2. The ^{1}H NMR (700.13 MHz) spectrum of cladoloside S (**1**) in C_5D_5N/D_2O (4/1)

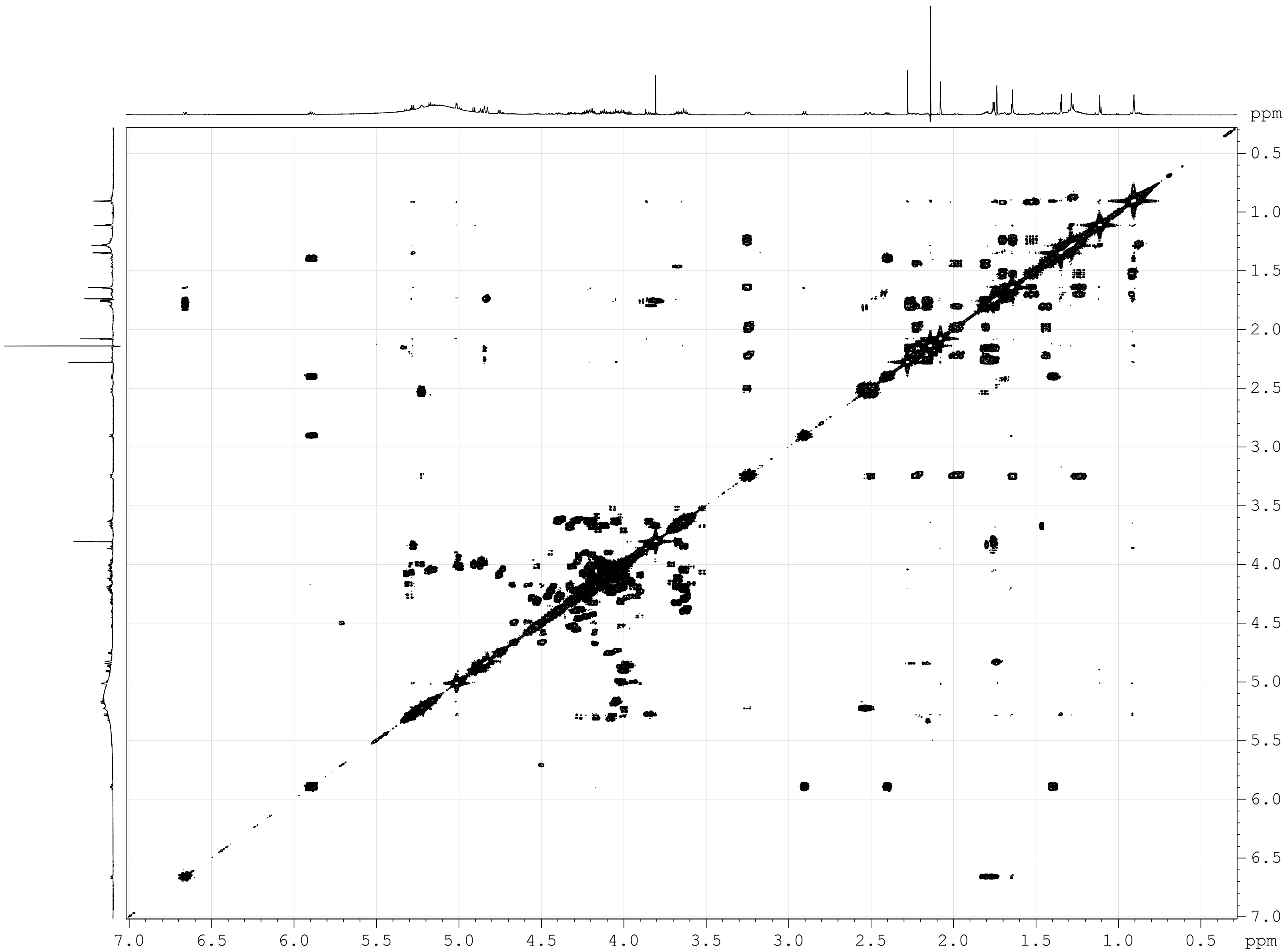

Figure S3. The COSY (700.13 MHz) spectrum of cladoloside S (**1**) in C_5D_5N/D_2O (4/1)

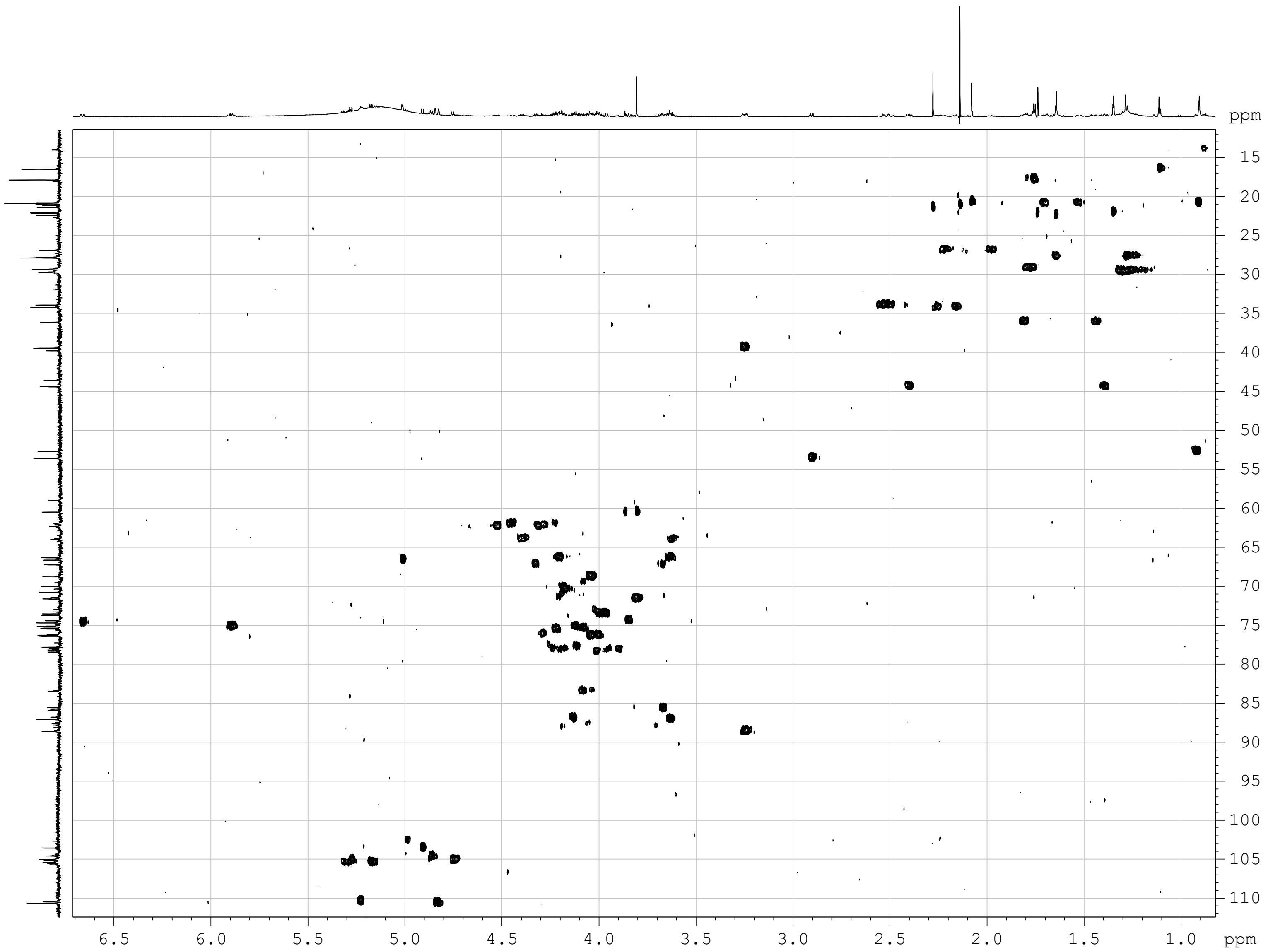

Figure S4. The HSQC (700.13 MHz) spectrum of cladoloside S (**1**) in C_5D_5N/D_2O (4/1)

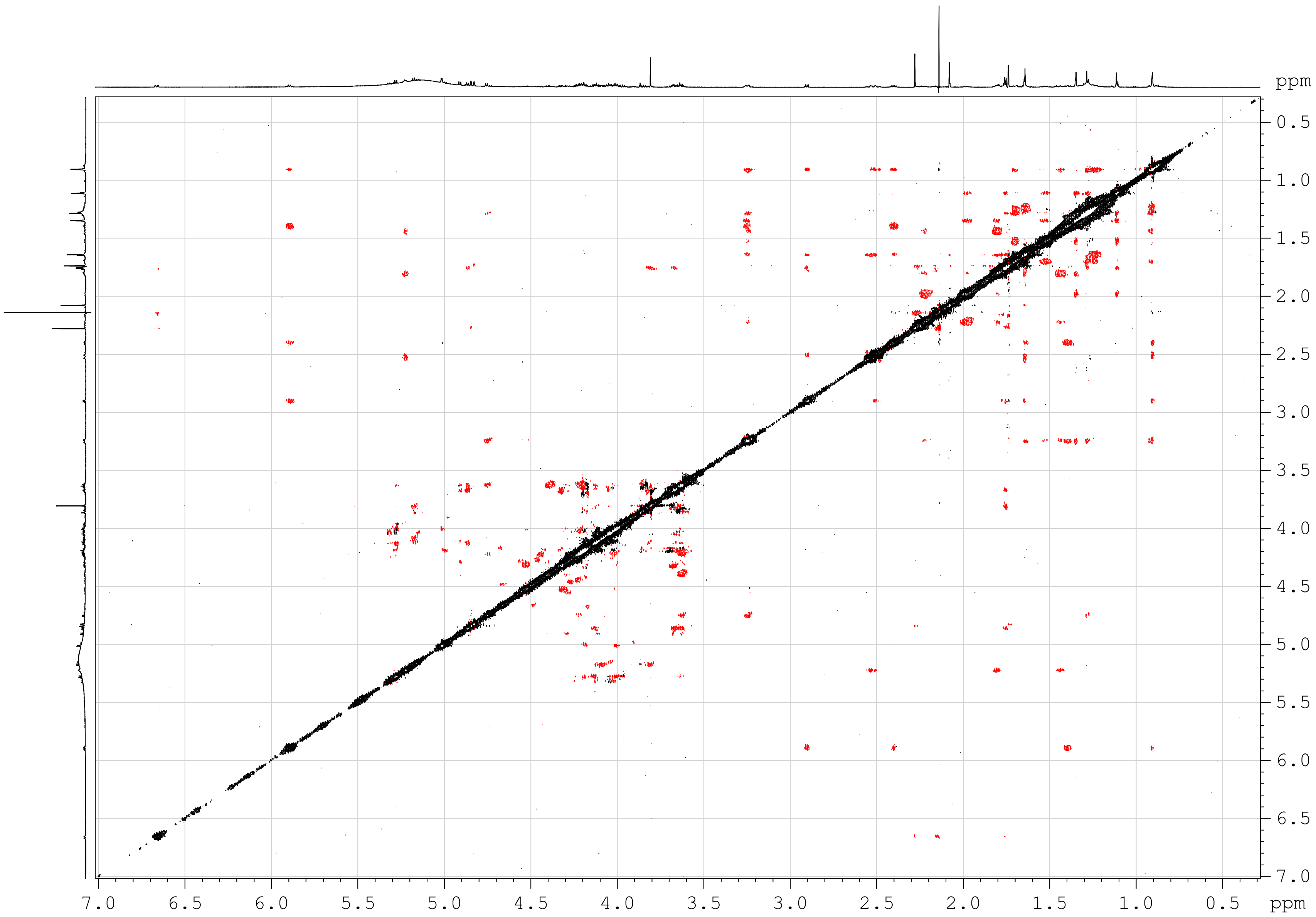

Figure S5. The ROESY (700.13 MHz) spectrum of cladoloside S (**1**) in C_5D_5N/D_2O (4/1)

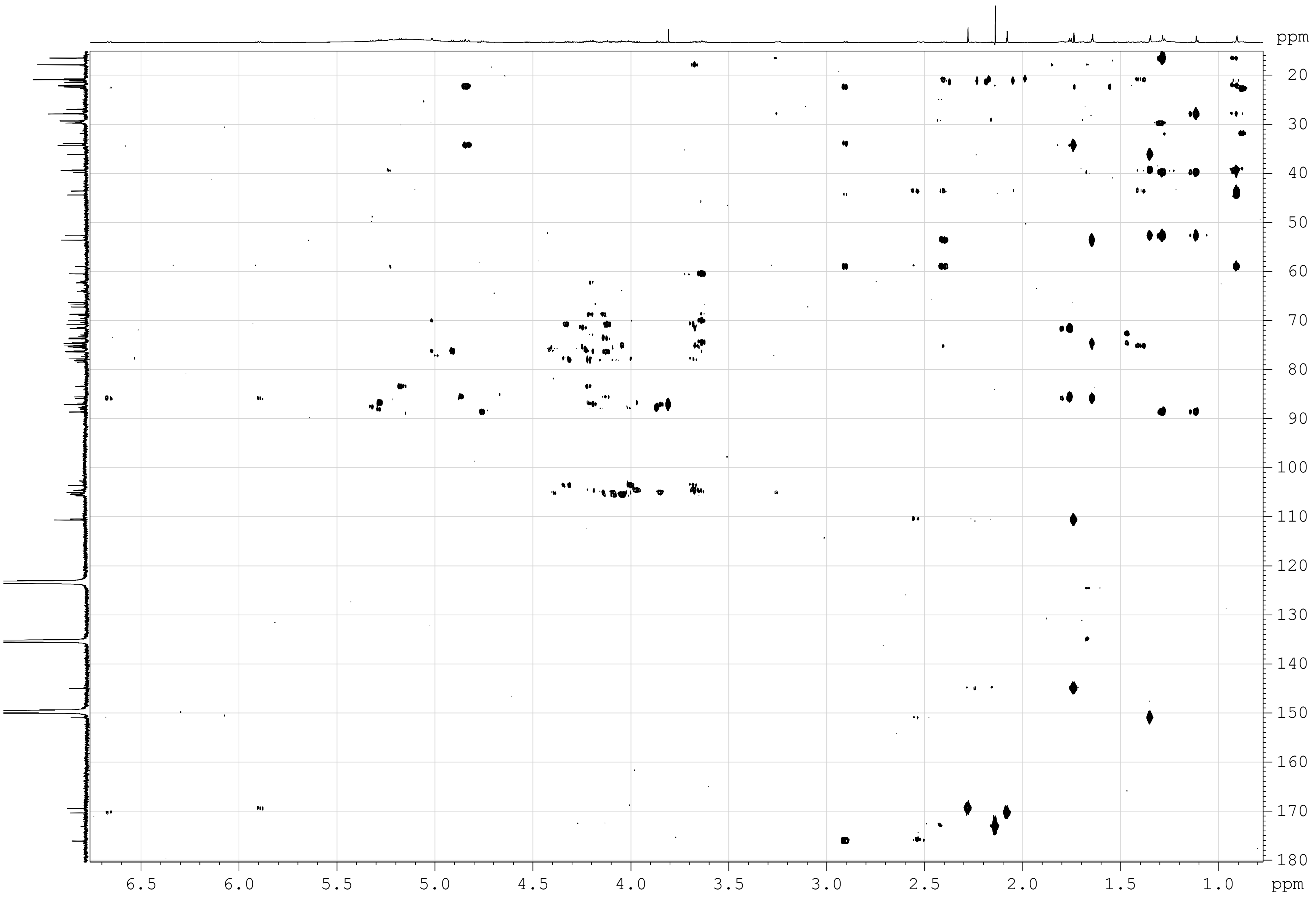

Figure S6. The HMBC (700.13 MHz) spectrum of cladoloside S (**1**) in C_5D_5N/D_2O (4/1)

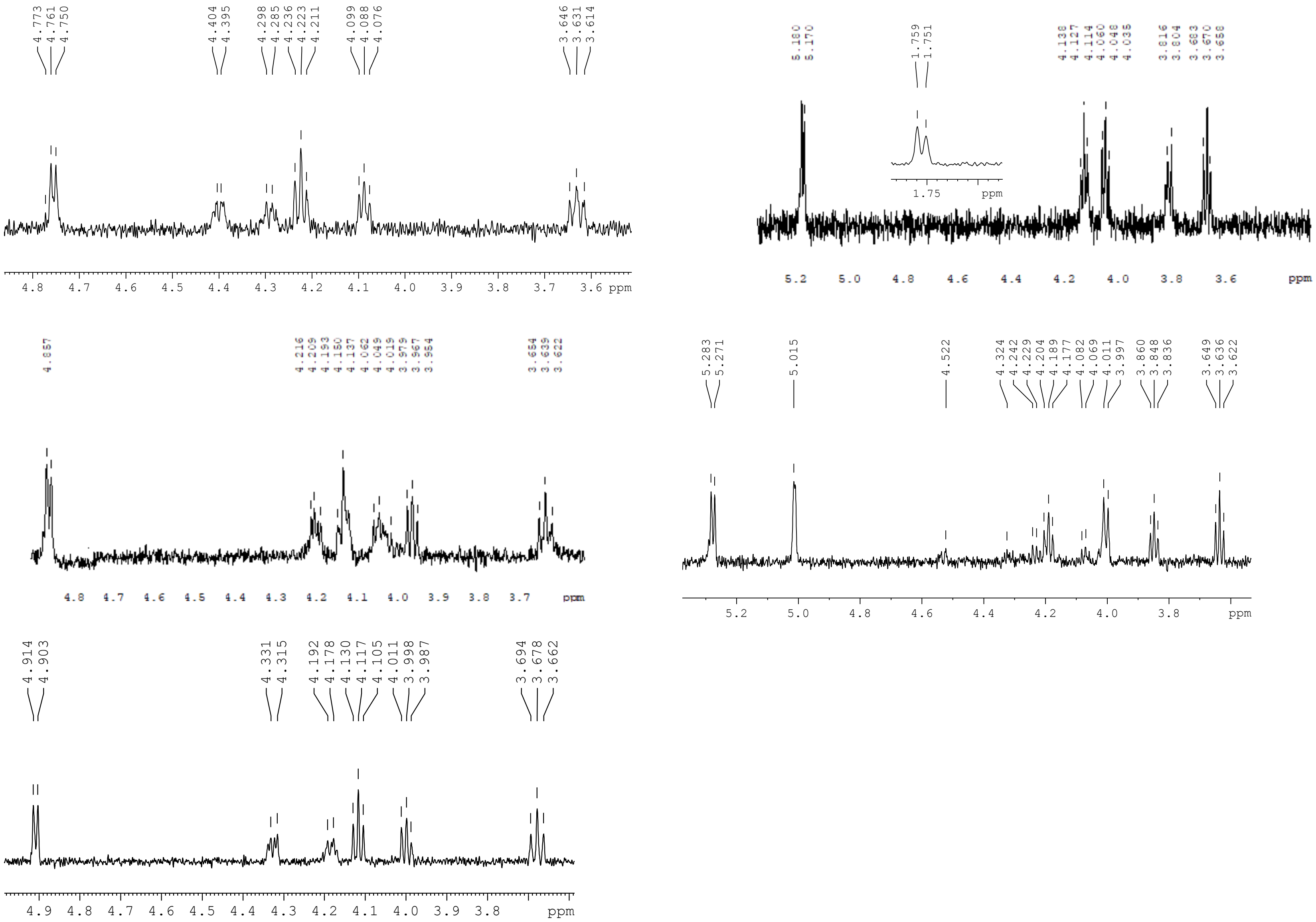

Figure S7. 1 D TOCSY (700.13 MHz) spectra of Xyl1, Qui2, Xyl3, MeGlc4, Xyl5 of cladoloside S (**1**) in C_5D_5N/D_2O (4/1)

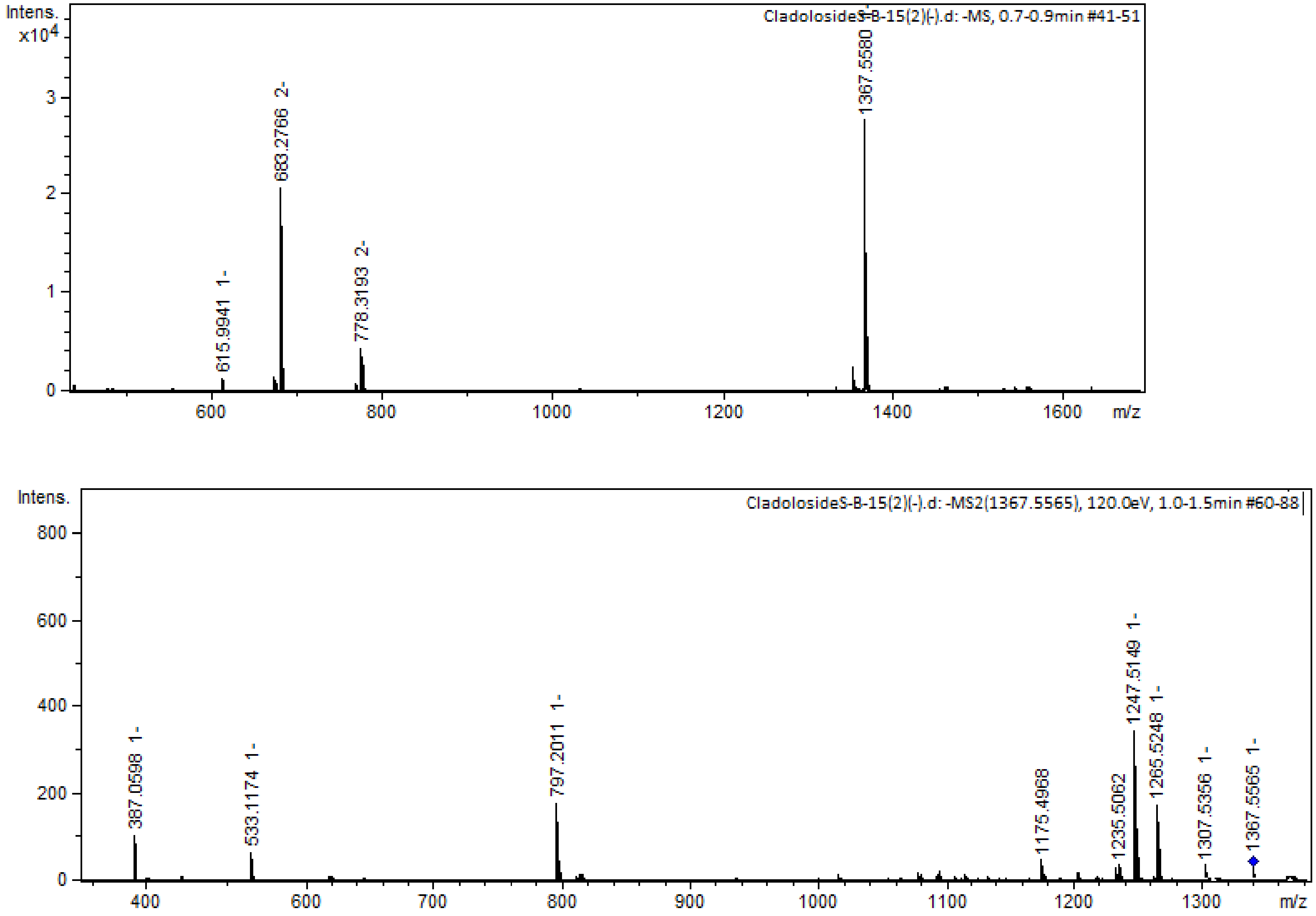

Figure S8. (–)HR-ESI-MS and (–)HR-ESI-MS/MS spectra of cladoloside S (**1**)

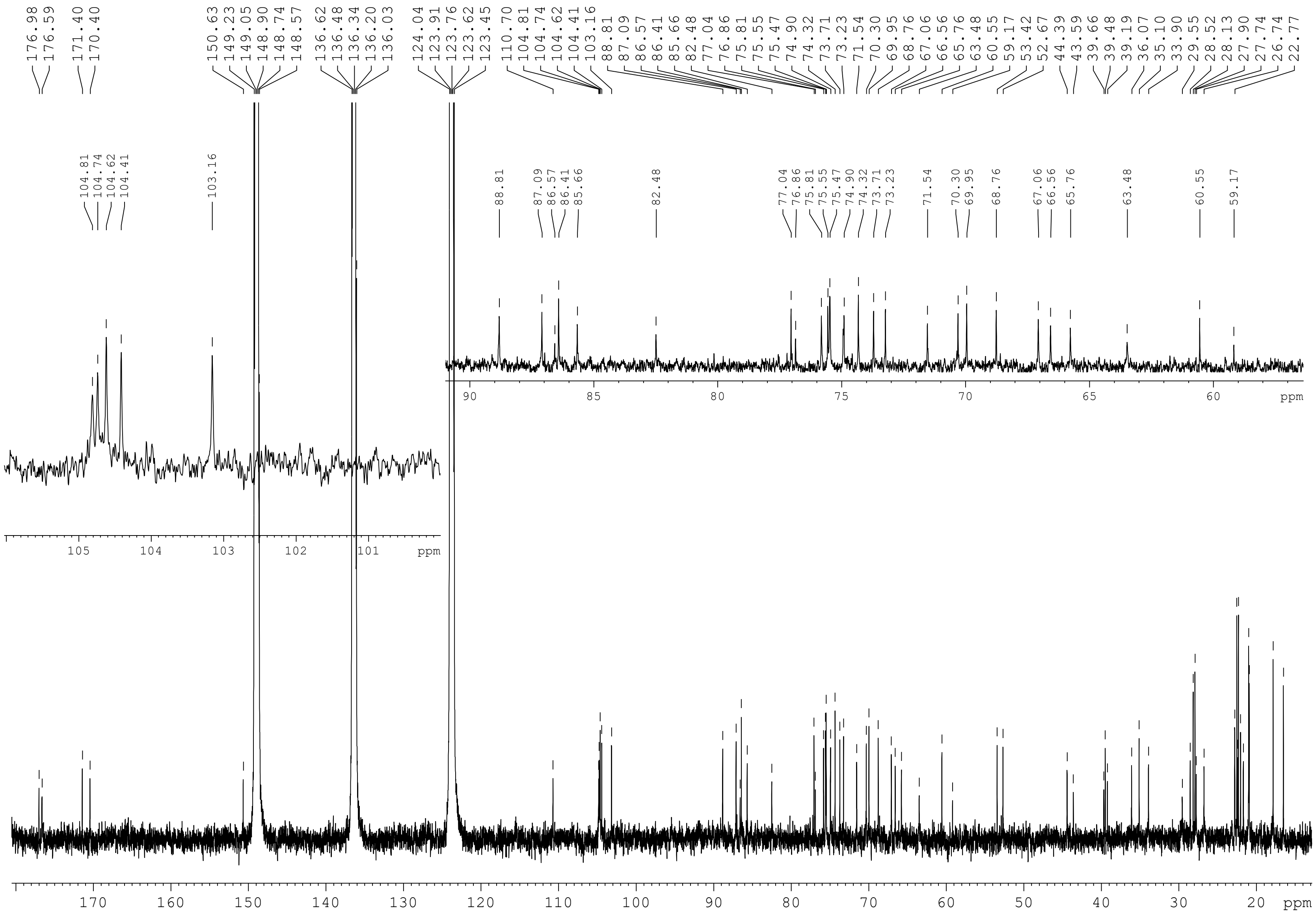

Figure S9. The ^{13}C NMR (176.04 MHz) spectrum of cladoloside S_1 (**2**) in C_5D_5N/D_2O (4/1)

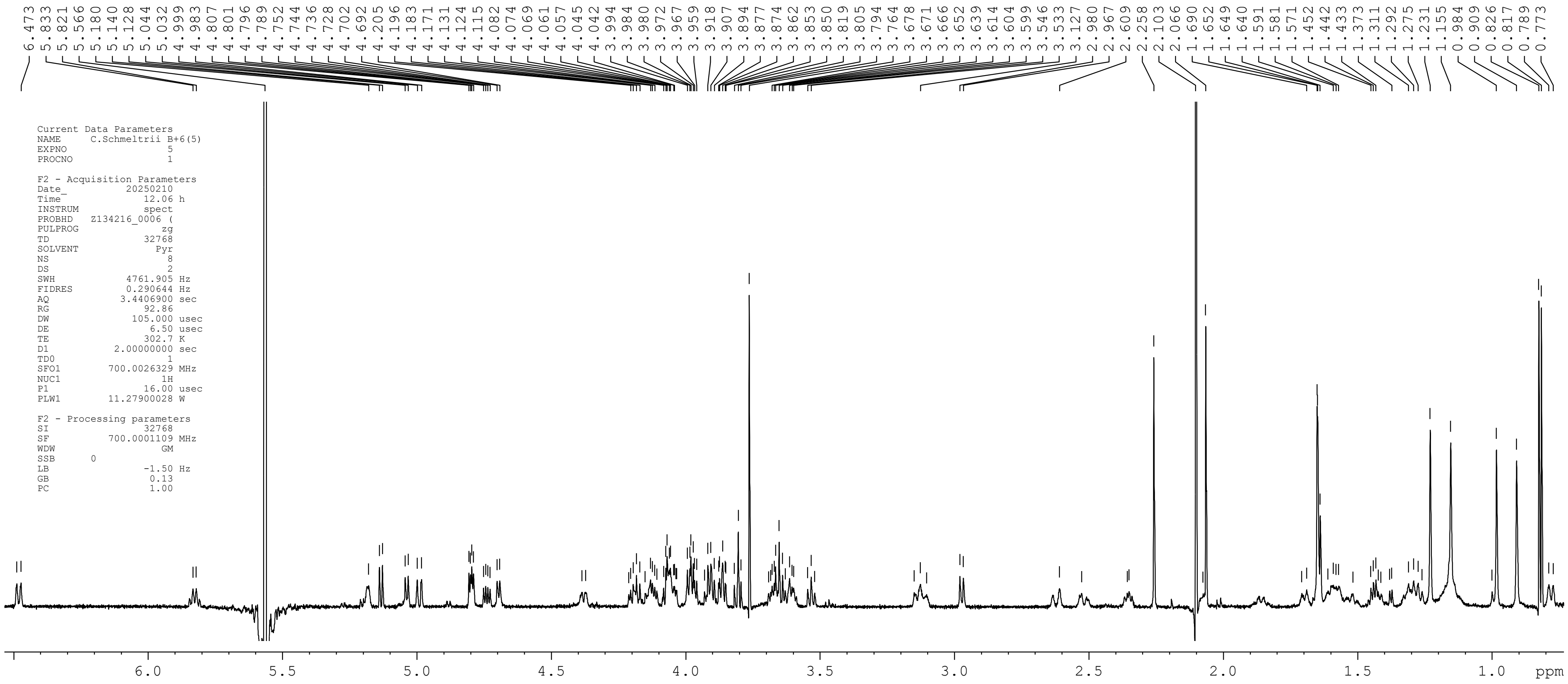

Figure S10. The ^{1}H NMR (700.13 MHz) spectrum of cladoloside S_1 (**2**) in C_5D_5N/D_2O (4/1)

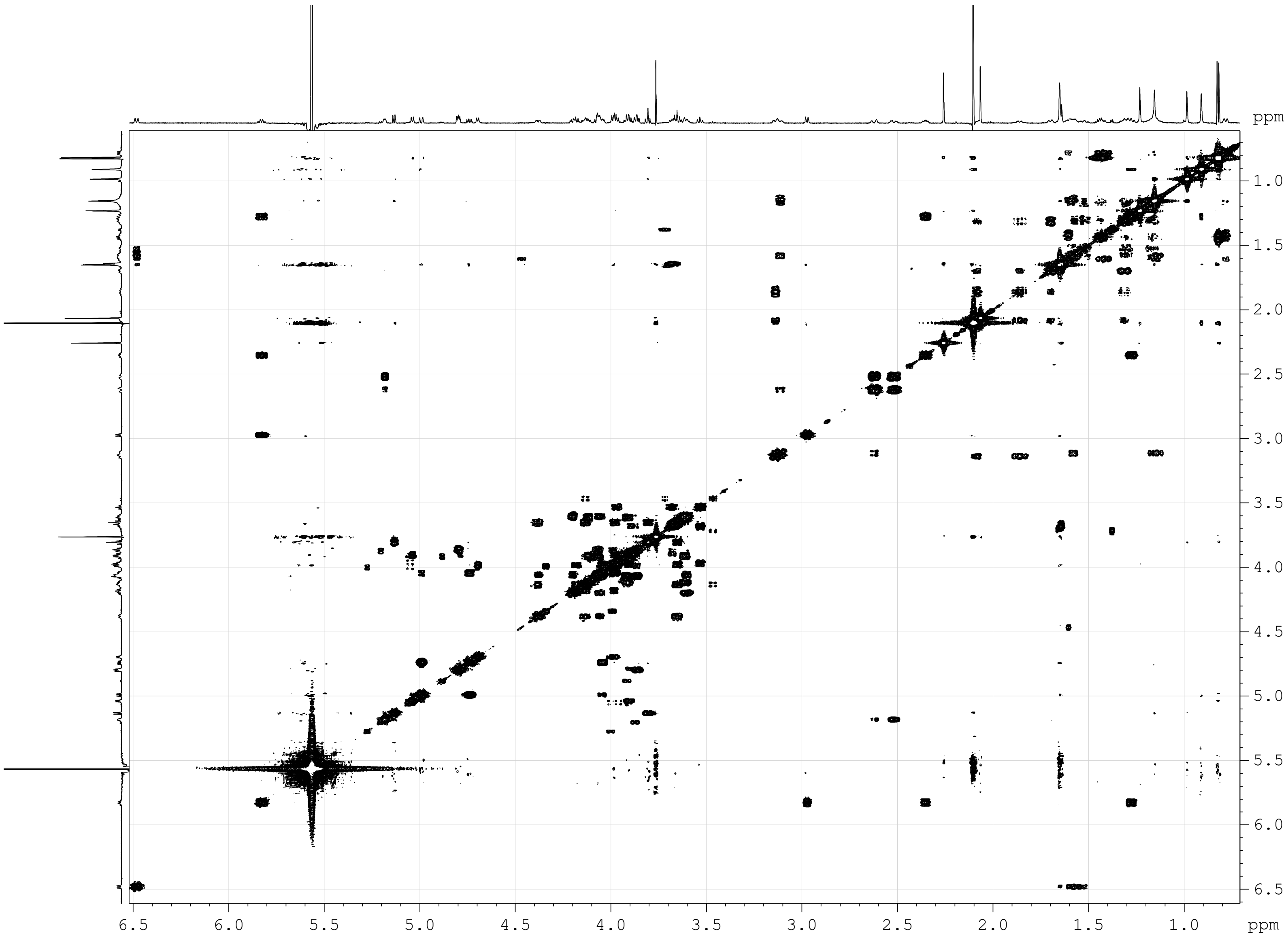

Figure S11. The COSY (700.13 MHz) spectrum of cladoloside S_1 (**2**) in C_5D_5N/D_2O (4/1)

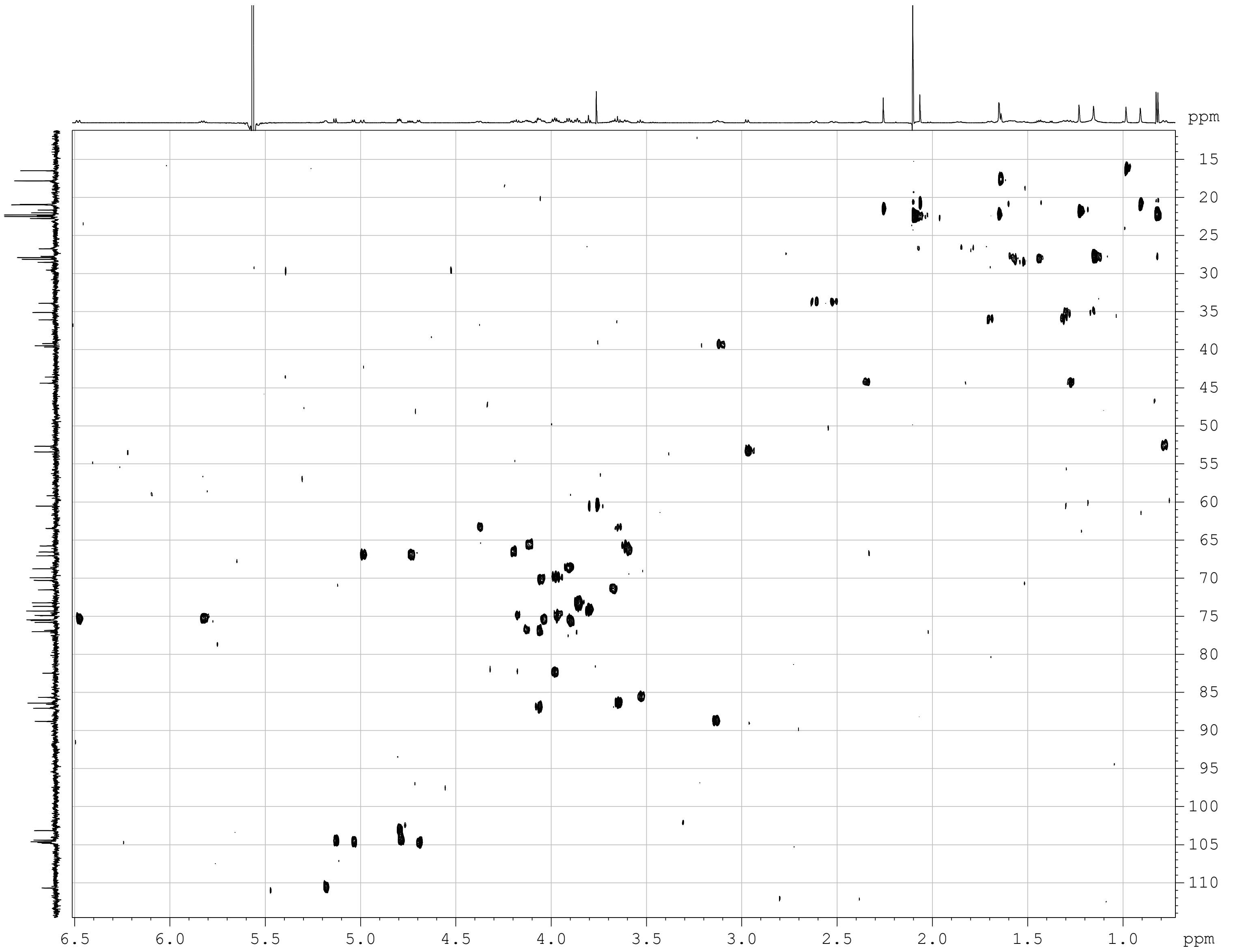

Figure S12. The HSQC (700.13 MHz) spectrum of cladoloside S_1 (**2**) in C_5D_5N/D_2O (4/1)

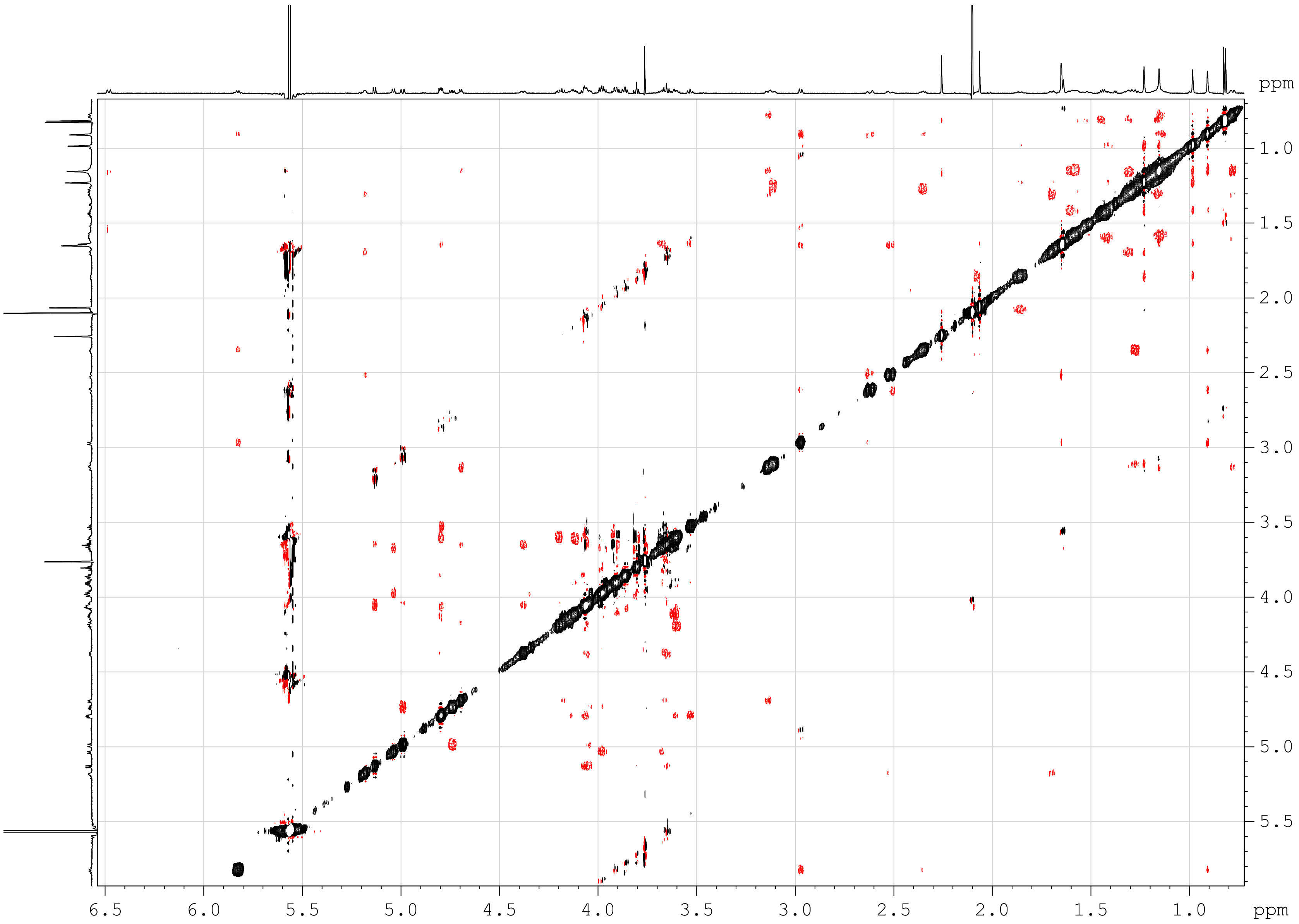

Figure S13. The ROESY (700.13 MHz) spectrum of cladoloside S_1 (**2**) in C_5D_5N/D_2O (4/1)

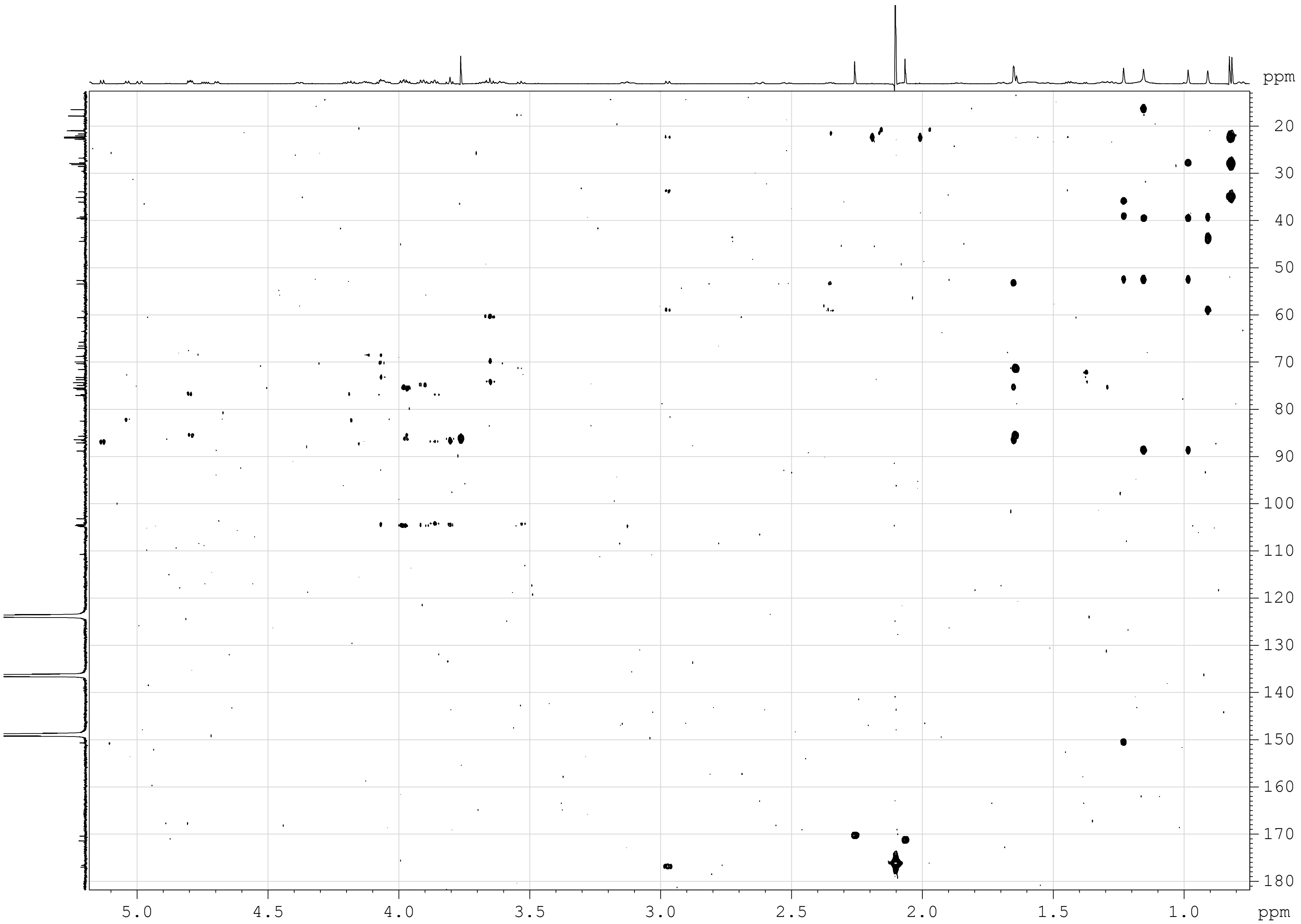

Figure S14. The HMBC (700.13 MHz) spectrum of cladoloside S1 (**2**) in C_5D_5N/D_2O (4/1)

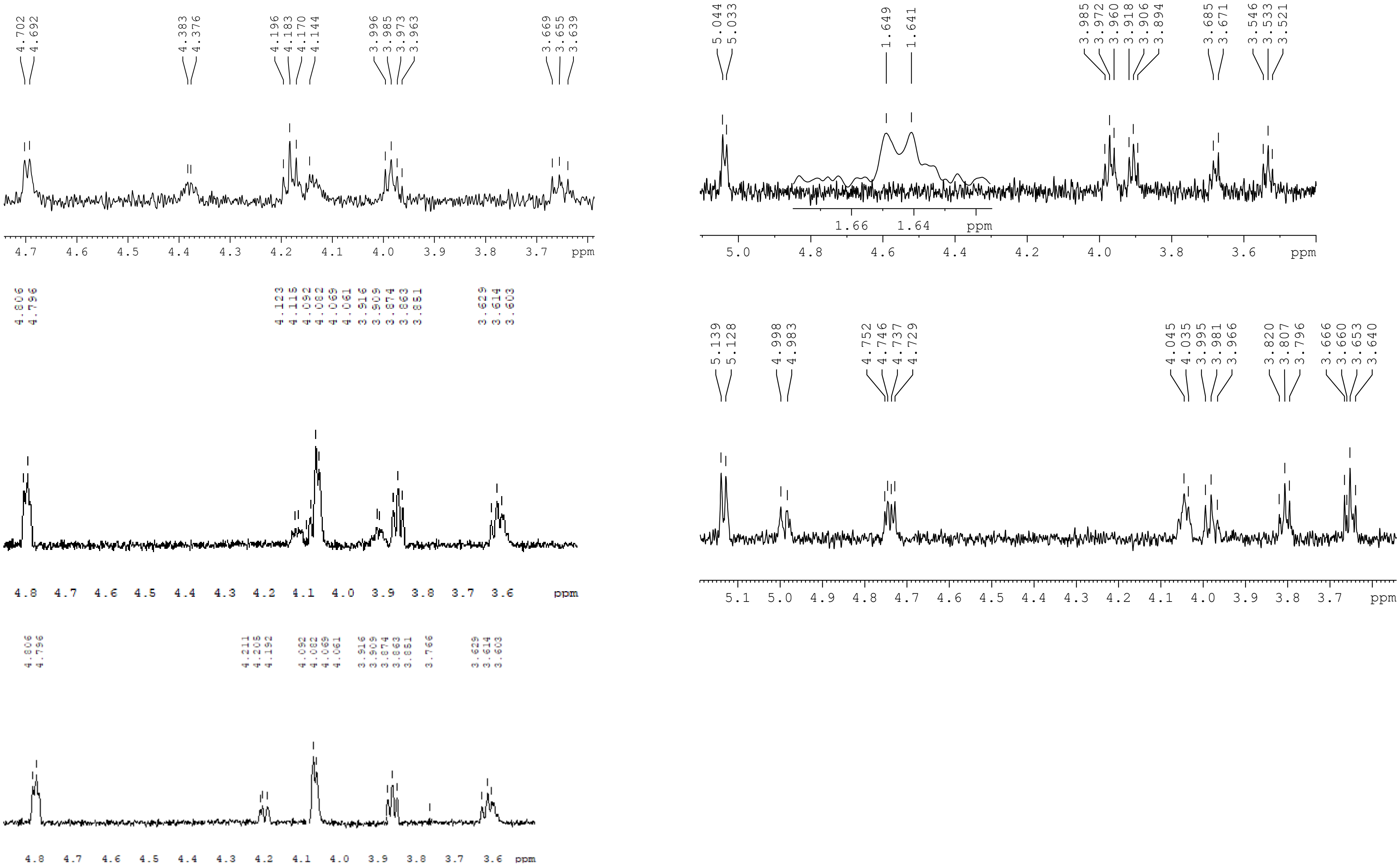

Figure S15. 1 D TOCSY (700.13 MHz) spectra of Xyl1, Qui2, Xyl3, MeGlc4, Xyl5 of cladoloside S_1 (**2**) in C_5D_5N/D_2O (4/1)

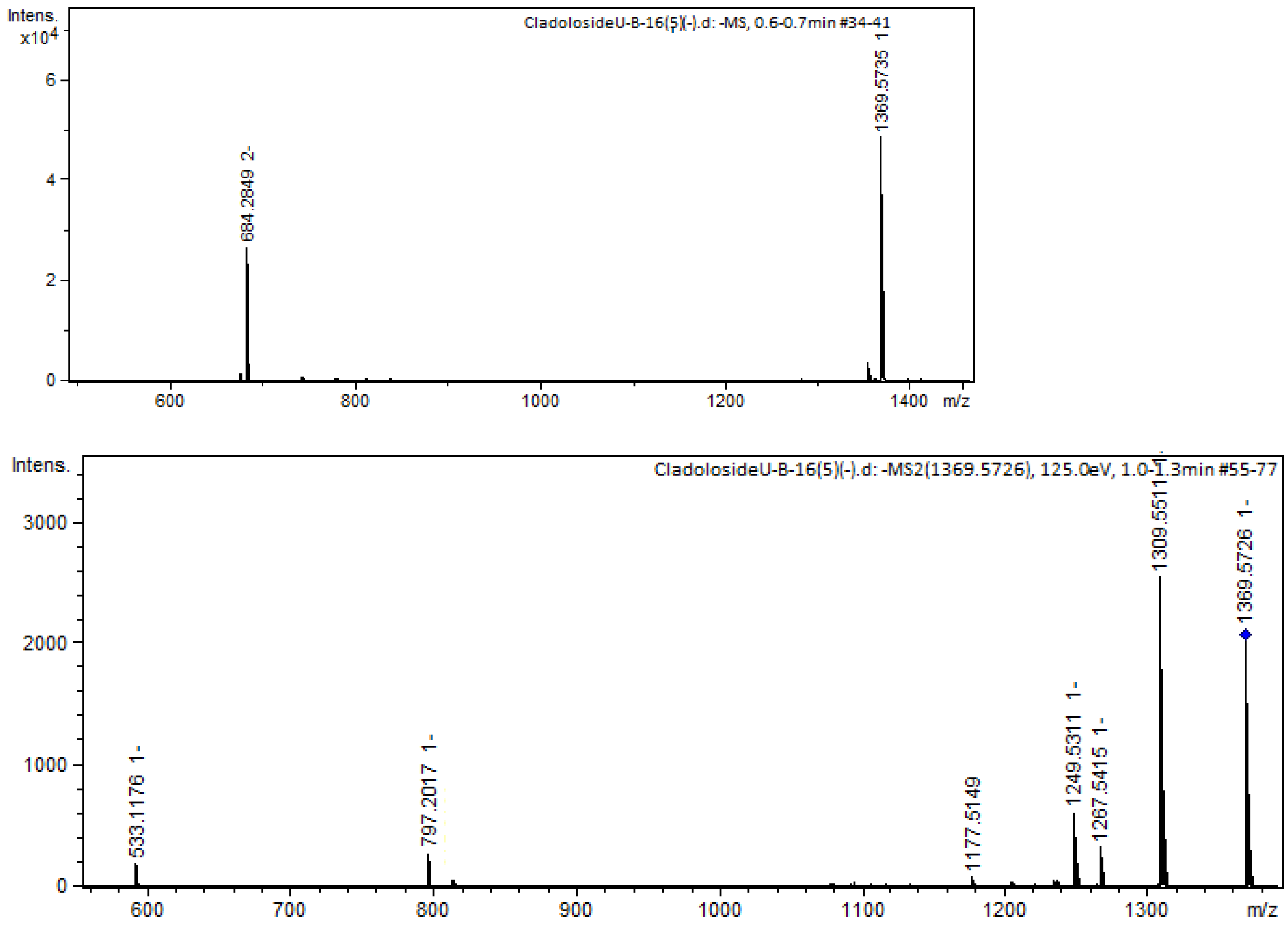

Figure S16. (–)HR-ESI-MS and (–)HR-ESI-MS/MS spectra of cladoloside S_1 (**2**)

Table S1. One- and two-dimensional NMR data of the aglycone moiety of cladoloside S_1 (**2**).

Position	δ_C, mult.[a]	δ_H, mult. (J in Hz)[b]	HMBC	ROESY
1	36.1, CH_2	1.70, m		H-19
		1.31, m		H-3, H-5
2	26.7, CH_2	2.07, m		
		1.85, m		H-19, H-30
3	88.8, CH	3.14, brd (11.7)	C:1 Xyl1	H-1, H-5, H-31, H1-Xyl1
4	39.7, C			
5	52.7, CH	0.78, brd (11.7)		H-3, H-31
6	21.0, CH_2	1.57, m		
		1.42, m		
7	27.7, CH_2	1.60, m		
		1.15, m		H-5, H-32
8	39.5, CH	3.12, brd (16.0)		H-15
9	150.6, C			
10	39.2, C			
11	110.7, CH	5.18, m		H-1
12	33.9, CH_2	2.62, brd (16.8)		
		2.52, dd (16.8; 6.0)	C: 17	H-21
13	59.2, C			
14	43.6, C			
15	44.4, CH_2	2.35, dd (12.0; 7.2)	C: 13, 17, 32	H-32
		1.28, brd (12.0)		H-8
16	75.5, CH	5.83, brq (9.6)		H-32
17	53.4, CH	2.97, d (9.6)	C: 12, 13, 18, 21	H-12, H-21, H-32
18	176.9, C			
19	22.0, CH_3	1.23, s	C: 1, 5, 9, 10	H-1, H-30
20	86.5, C			
21	22.5, CH_3	1.65, s	C: 17, 20, 22	
22	75.4, CH	6.48, d (10.8)		
23	28.5, CH_2	1.52, m		
24	35.1, CH_2	1.29, m		
		1.15, m		
25	28.1, CH	1.52, m		
26	22.5, CH_3	0.83, s	C: 24, 25, 27	
27	22.8, CH_3	0.82, s	C: 24, 25, 26	H-31
30	16.5, CH_3	0.98, s	C: 3, 4, 5, 31	H-2, H-6, H-19, H-30,
31	27.9, CH_3	1.16, s	C: 3, 4, 5, 30	H-1 Xyl1
32	21.0, CH_3	0.91, s	C: 8, 13, 14, 15	H-12, H-15, H-17, H-24
OAc-16	171.4, C			
	20.9, CH_3	2.07, s		
OAc-22	170.5, C			
	21.6, CH_3	2.25, s		H-22, H-24, H-26, H-27

[a] Recorded at 176.04 MHz in C_5D_5N/D_2O (4/1). [b] Recorded at 700.13 MHz in C_5D_5N/D_2O (4/1).

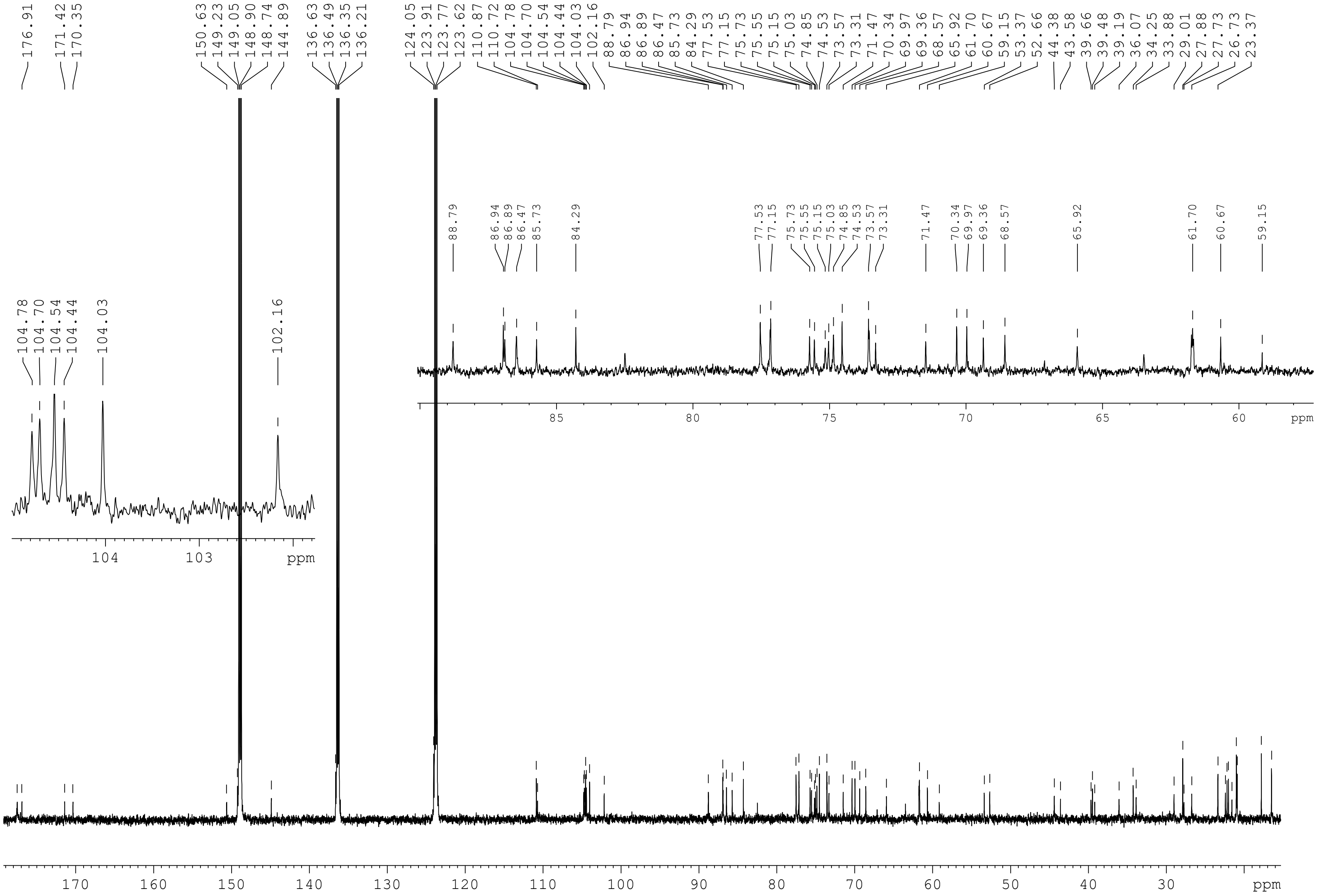

Figure S17. The ^{13}C NMR (176.04 MHz) spectrum of cladoloside T (**3**) in C_5D_5N/D_2O (4/1)

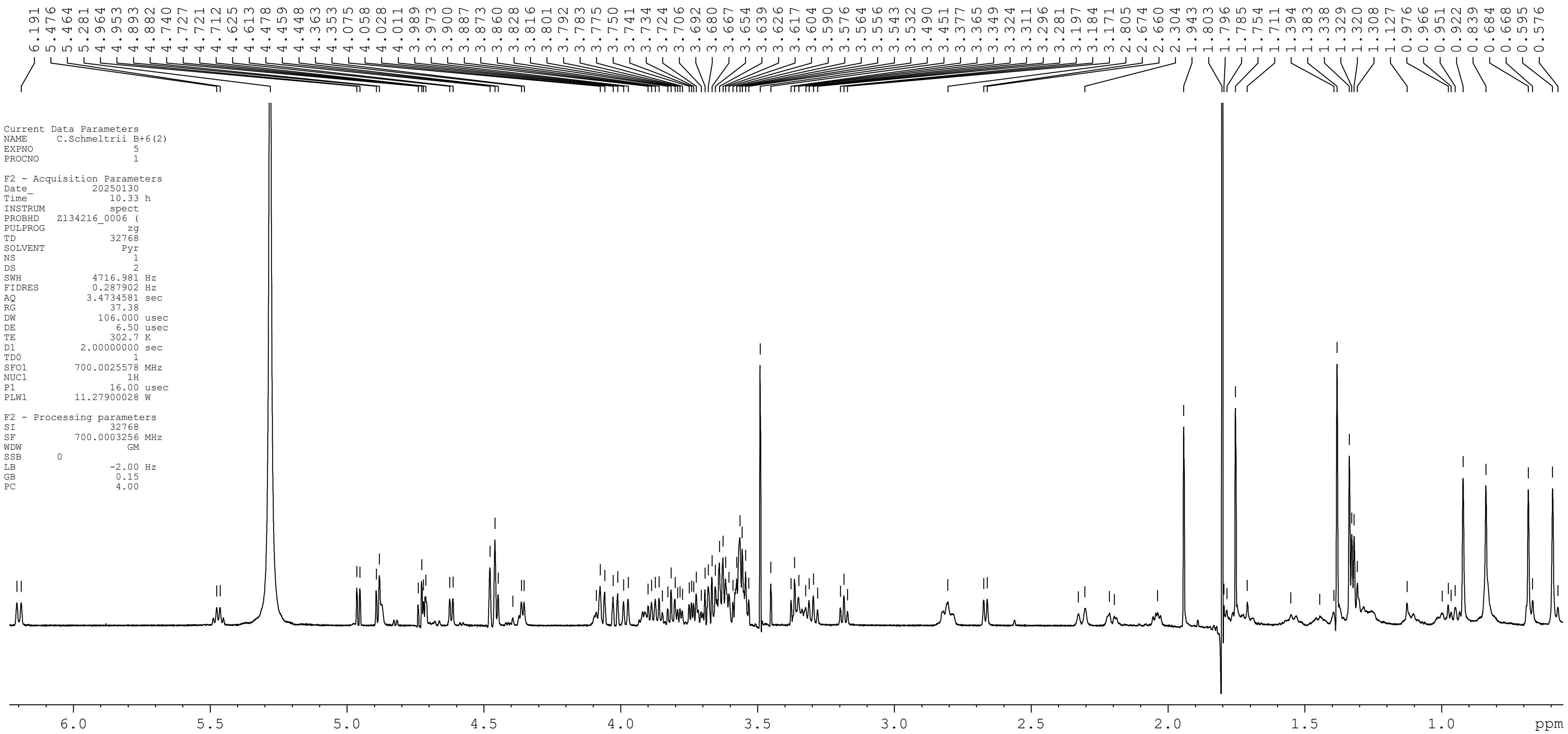

Figure S18. The ^{1}H NMR (700.13 MHz) spectrum of cladoloside T (**3**) in C_5D_5N/D_2O (4/1)

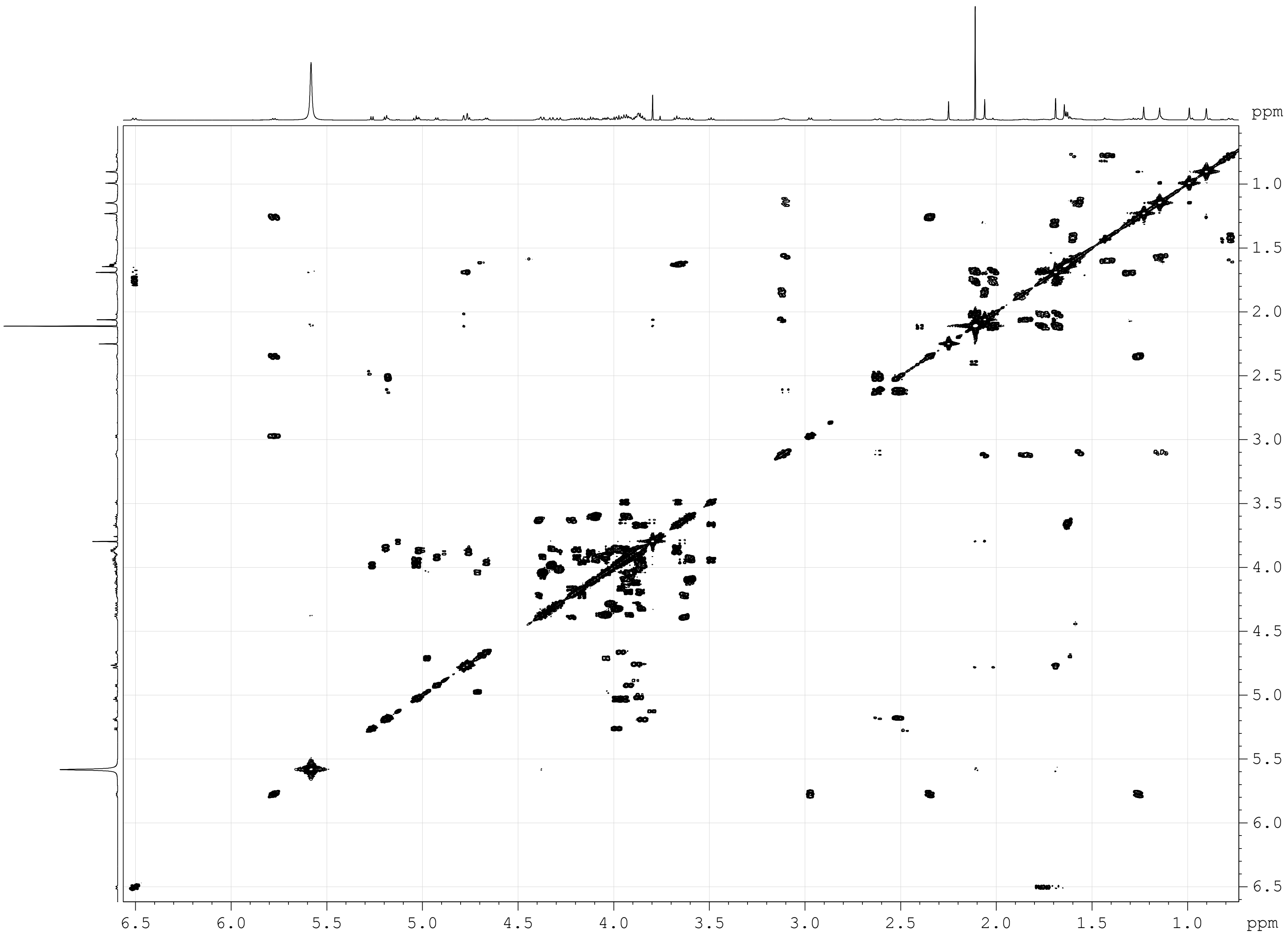

Figure S19. The COSY (700.13 MHz) spectrum of cladoloside T (**3**) in C_5D_5N/D_2O (4/1)

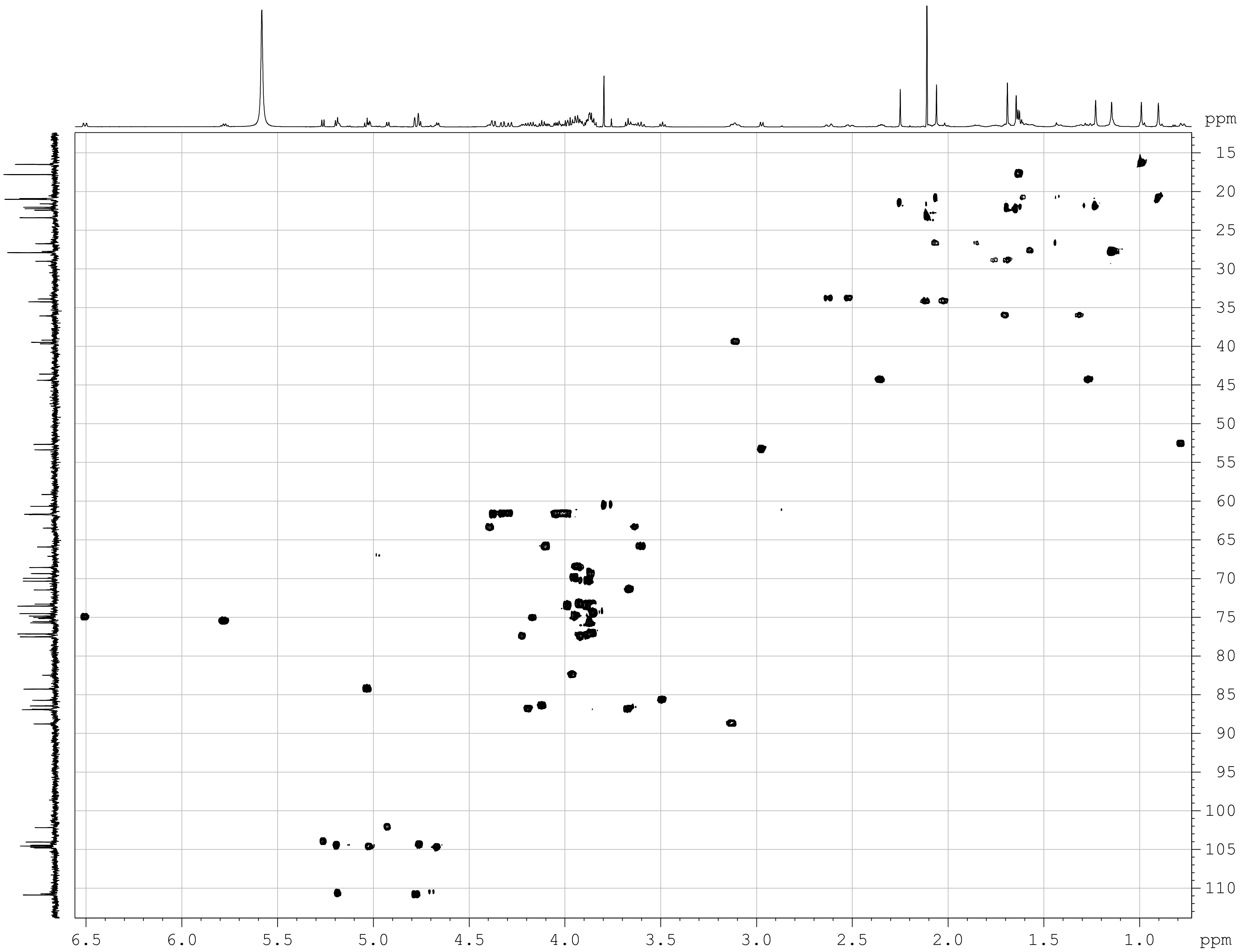

Figure S20. The HSQC (700.13 MHz) spectrum of cladoloside T (**3**) in C_5D_5N/D_2O (4/1)

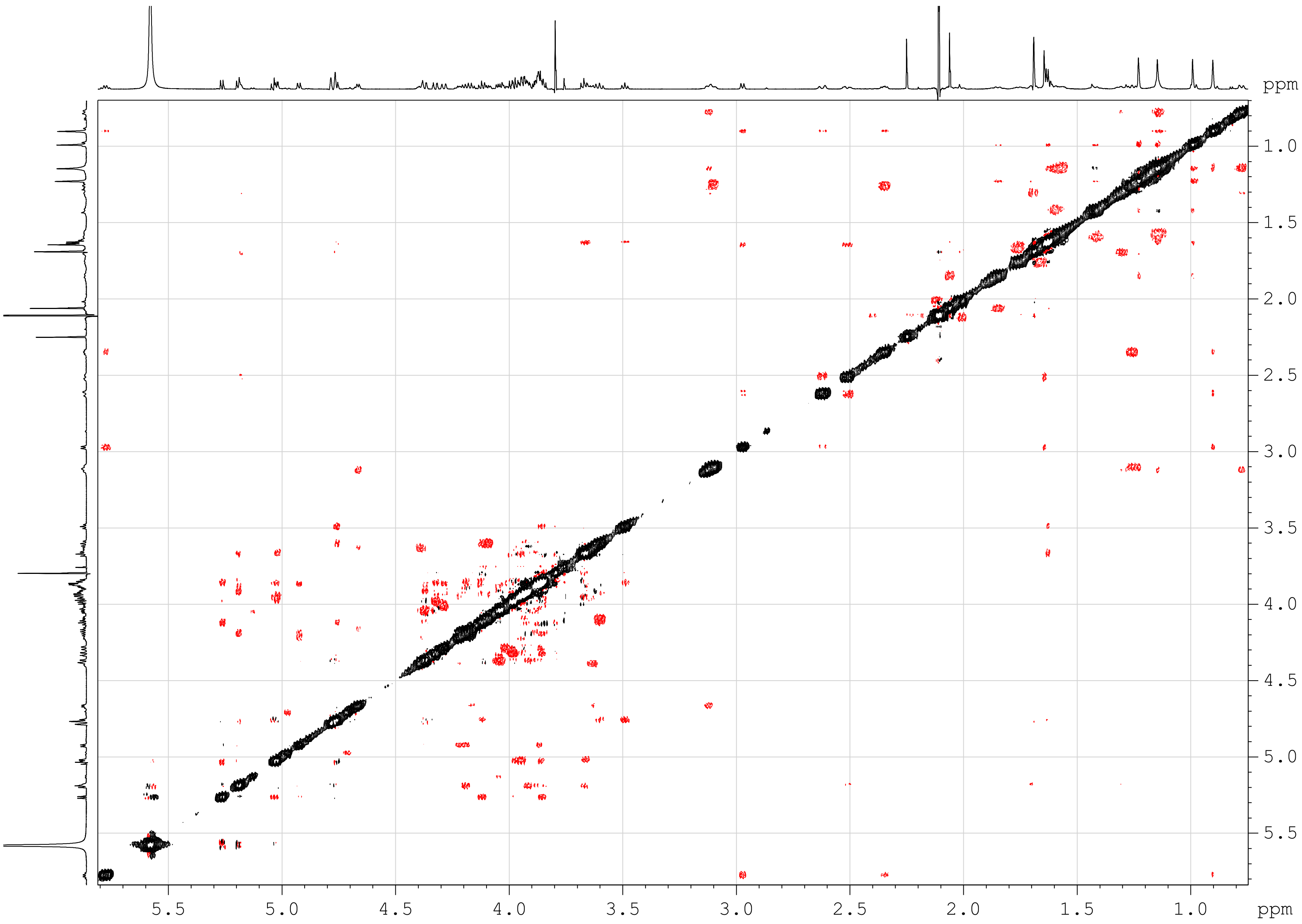

Figure S21. The ROESY (700.13 MHz) spectrum of cladoloside T (**3**) in C_5D_5N/D_2O (4/1)

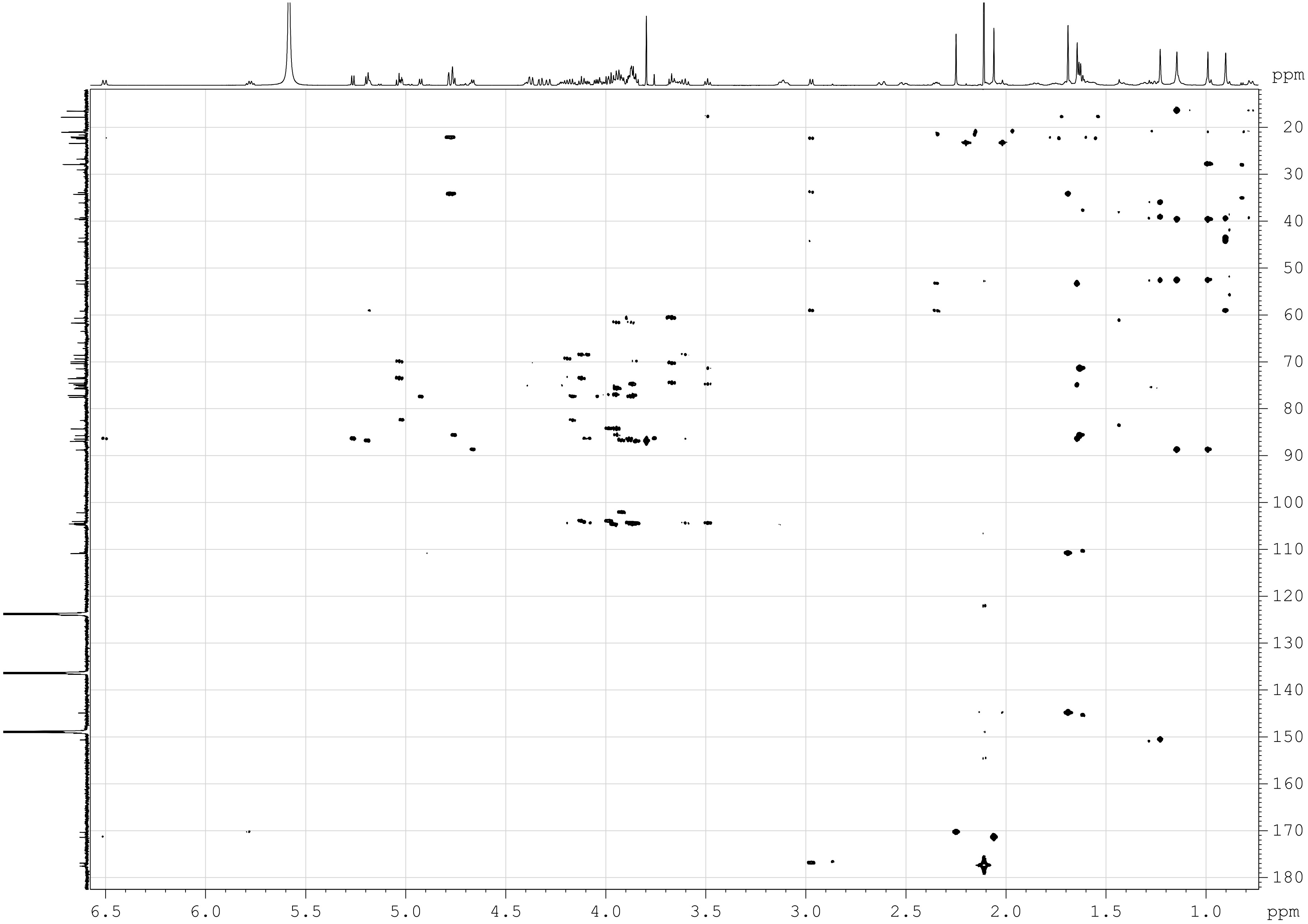

Figure S22. The HMBC (700.13 MHz) spectrum of cladoloside T (**3**) in C_5D_5N/D_2O (4/1)

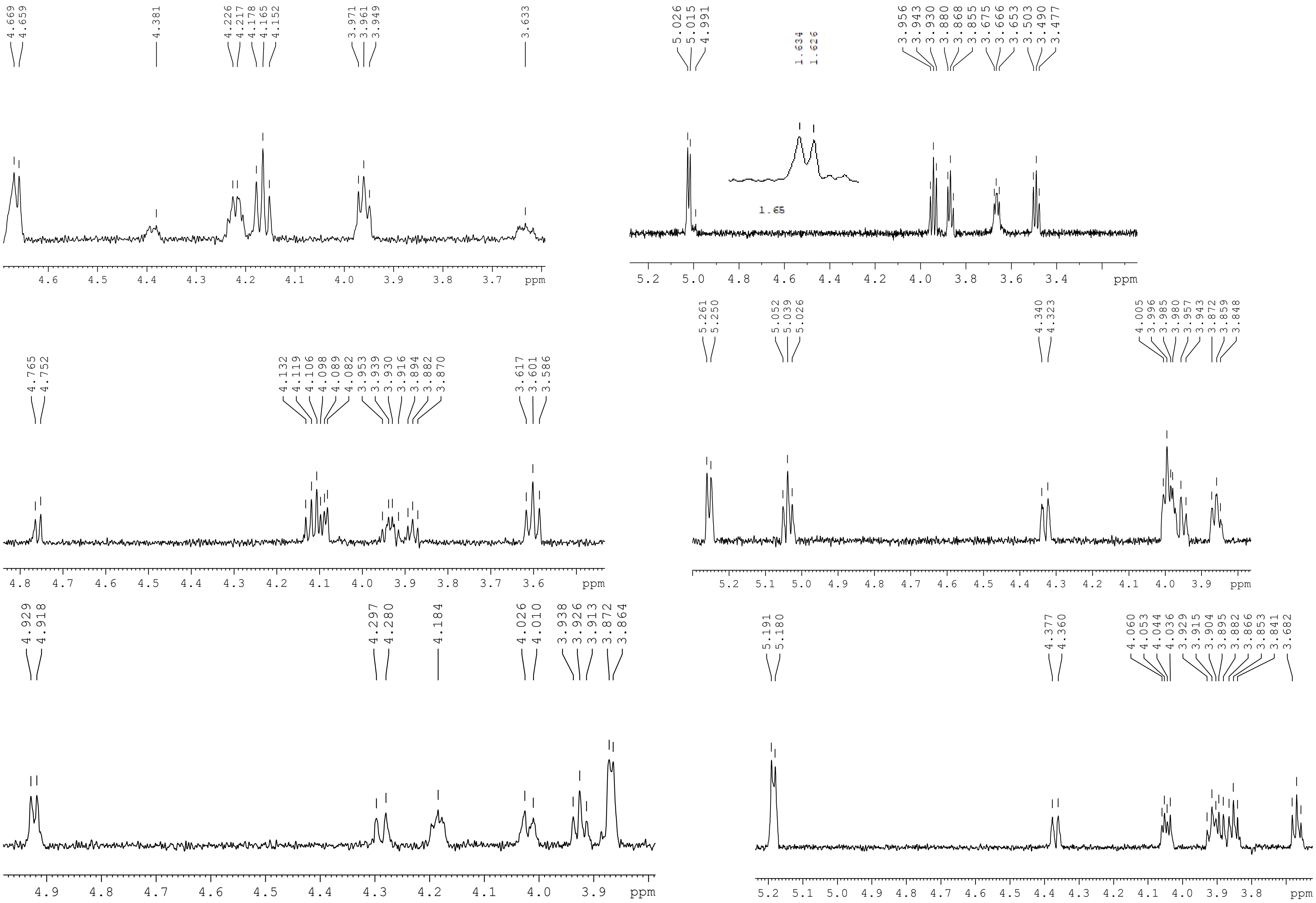

Figure S23. 1 D TOCSY (700.13 MHz) spectra of Xyl1, Qui2, Xyl3, Glc4, Glc5, MeGlc6 of cladoloside T (**3**) in C_5D_5N/D_2O (4/1)

Table S2. One- and two-dimensional NMR data of the aglycone moiety of cladoloside T (**3**).

Position	δ_C, mult.[a]	δ_H, mult. (J in Hz)[b]	HMBC	ROESY
1	36.1, CH_2	1.71, m		H-11
		1.31, m	C: 19	H-3, H-5, H-11
2	26.7, CH_2	2.07, m		
		1.85, m		H-19, H-30
3	88.8, CH	3.12, m	C: 4, 30, 31, C:1 Xyl1	H-1, H-5, H-31, H1-Xyl1
4	39.7, C			
5	52.7, CH	0.78, brd (13.1)	C: 4, 19, 30, 31	H-1, H-3, H-31
6	20.9, CH_2	1.60, m	C: 7, 19	
		1.43, m		H-8, H-19, H-30
7	27.7, CH_2	1.57, m		H-15, H-32
		1.12, m		
8	39.5, CH	3.11, brd (14.0)		H-6, H-15, H-19
9	150.6, C			
10	39.2, C			
11	110.7, CH	5.18, m	C: 10, 12, 13	
12	33.9, CH_2	2.62, brd (17.2)	C: 9, 11, 13, 18	H-17, H-32
		2.52, dd (17.2; 5.7)	C: 9, 11, 13, 14, 18	H-21
13	59.1, C			
14	43.6, C			
15	44.4, CH_2	2.35, dd (11.4; 6.5)	C: 13, 14, 16, 17, 32	H-7, H-32
		1.27, brd (11.4)	C: 14, 16, 32	H-8
16	75.6, CH	5.78, brq (9.0)	C: 13, 15, 17, 20, OAc-16	
17	53.4, CH	2.97, d (9.0)	C: 12, 13, 15, 16, 18, 20, 21	H-12, H-21, H-32
18	176.9, C			
19	22.0, CH_3	1.23, s	C: 1, 5, 9, 10	H-1, H-2, H-6, H-8, H-30
20	86.4, C			
21	22.4, CH_3	1.64, s	C: 17, 20, 22	H-12, H-17
22	75.0, CH	6.50, d (11.4)	C: 20, 21, 23, 24, OAc-22	
23	29.0, CH_2	1.76, m		
		1.69, m		
24	34.2, CH_2	2.12, m	C: 23, 25, 26, 27	
		2.02, m	C: 22, 23, 25, 26, 27	H-22
25	144.9, C			
26	110.9, CH_2	4.78, brs	C: 24, 25, 27	
		4.77, brs	C: 24, 25, 27	
27	22.2, CH_3	1.69, s	C: 24, 25, 26	H-26
30	16.5, CH_3	0.98, s	C: 3, 4, 5, 31	H-2, H-6, H-19, H-31
31	27.9, CH_3	1.15, s	C: 3, 4, 5, 30	H-3, H-5, H-6, H-30, H-1Xyl1
32	21.0, CH_3	0.91, s	C: 8, 13, 14, 15	H-7, H-12, H-15, H-16, H-17
OAc-16	170.3, C			
	21.5, CH_3	2.25, s	C: 16	H-22
OAc-22	171.4, C			
	20.9, CH_3	2.07, s	C:22	

[a] Recorded at 176.04 MHz in C_5D_5N/D_2O (4/1). [b] Recorded at 700.13 MHz in C_5D_5N/D_2O (4/1).

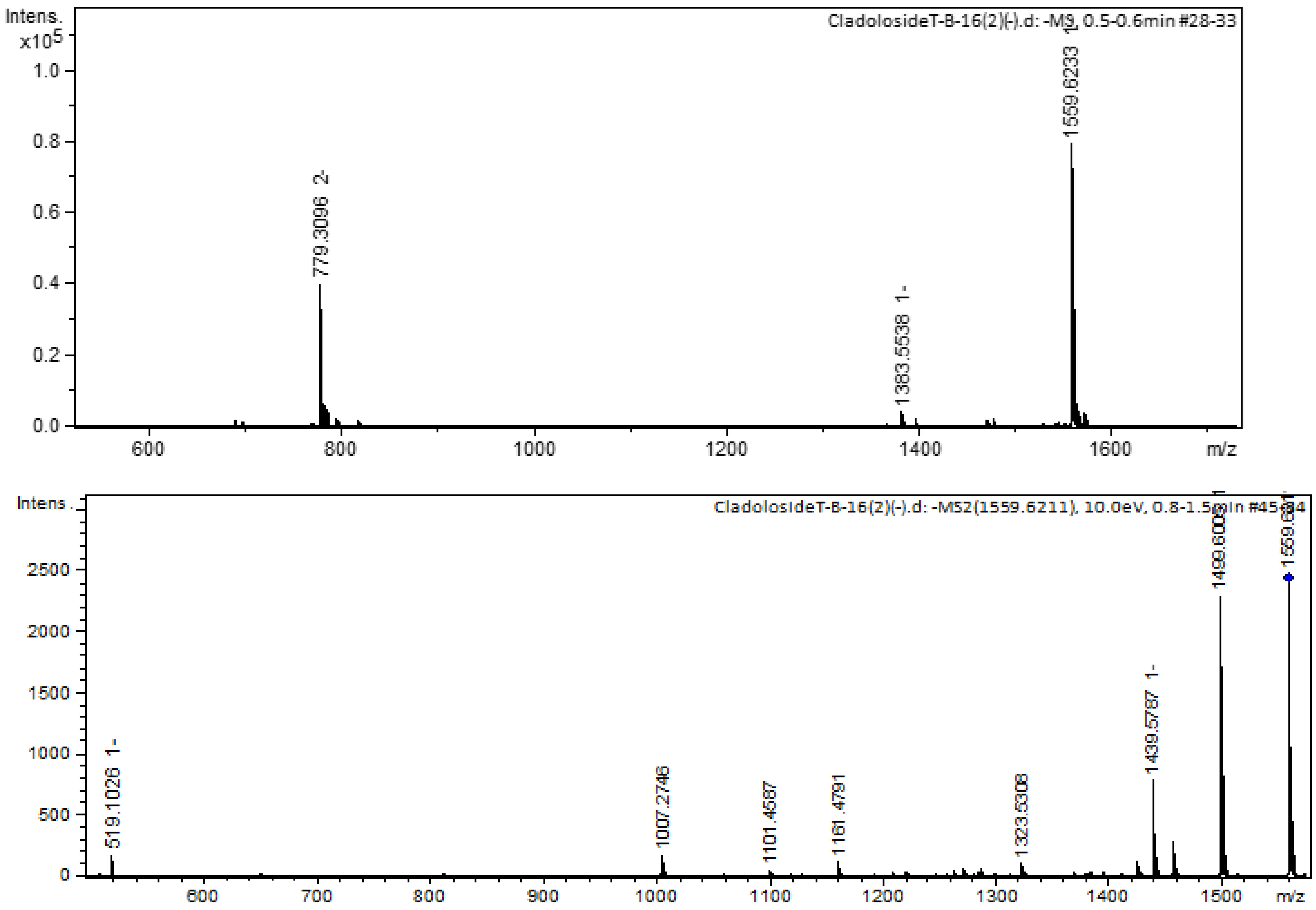

Figure S24. (–)HR-ESI-MS and (–)HR-ESI-MS/MS spectra of cladoloside T (**3**)

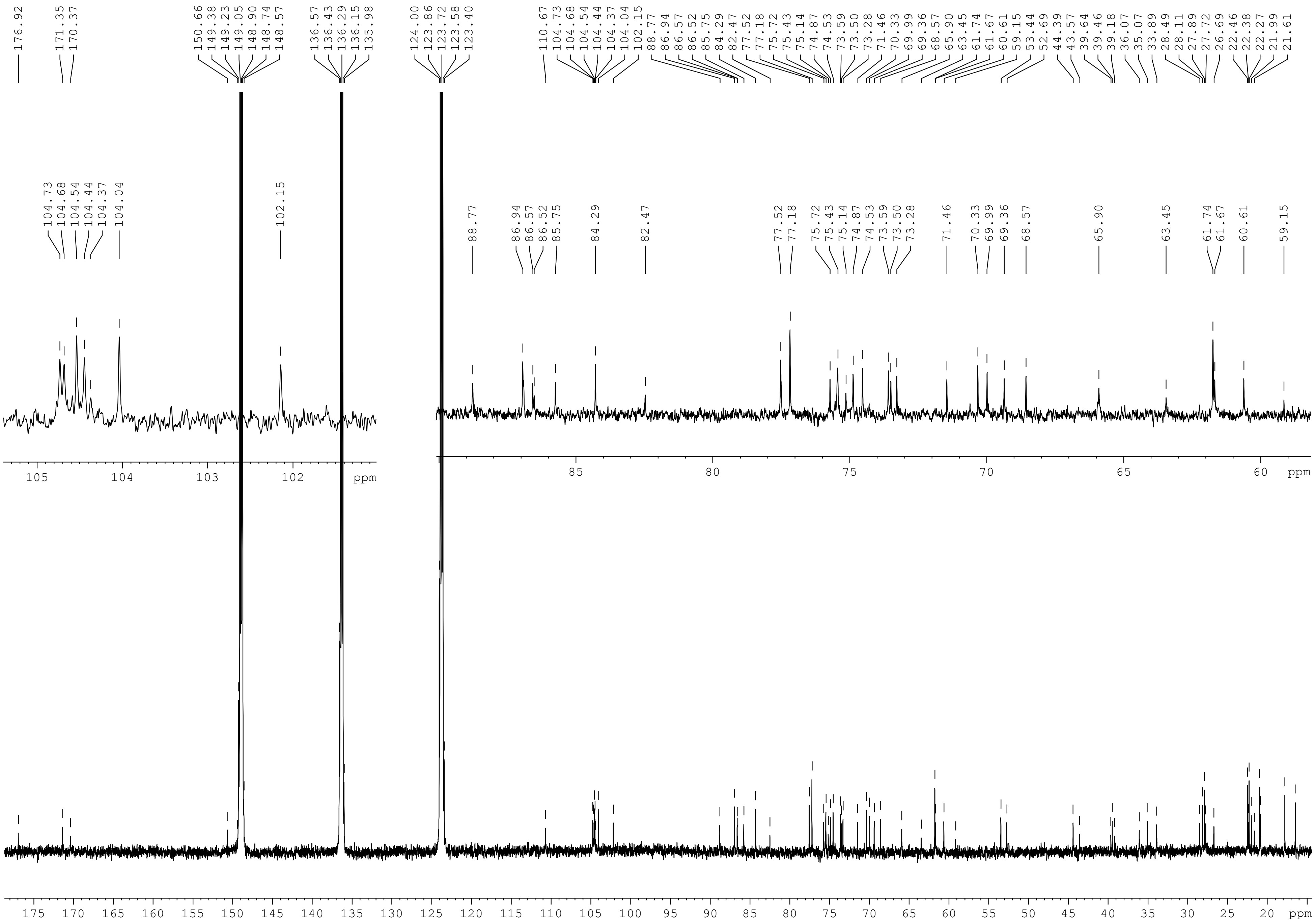

Figure S25. The ^{13}C NMR (176.04 MHz) spectrum of cladoloside T_1 (**4**) in C_5D_5N/D_2O (4/1)

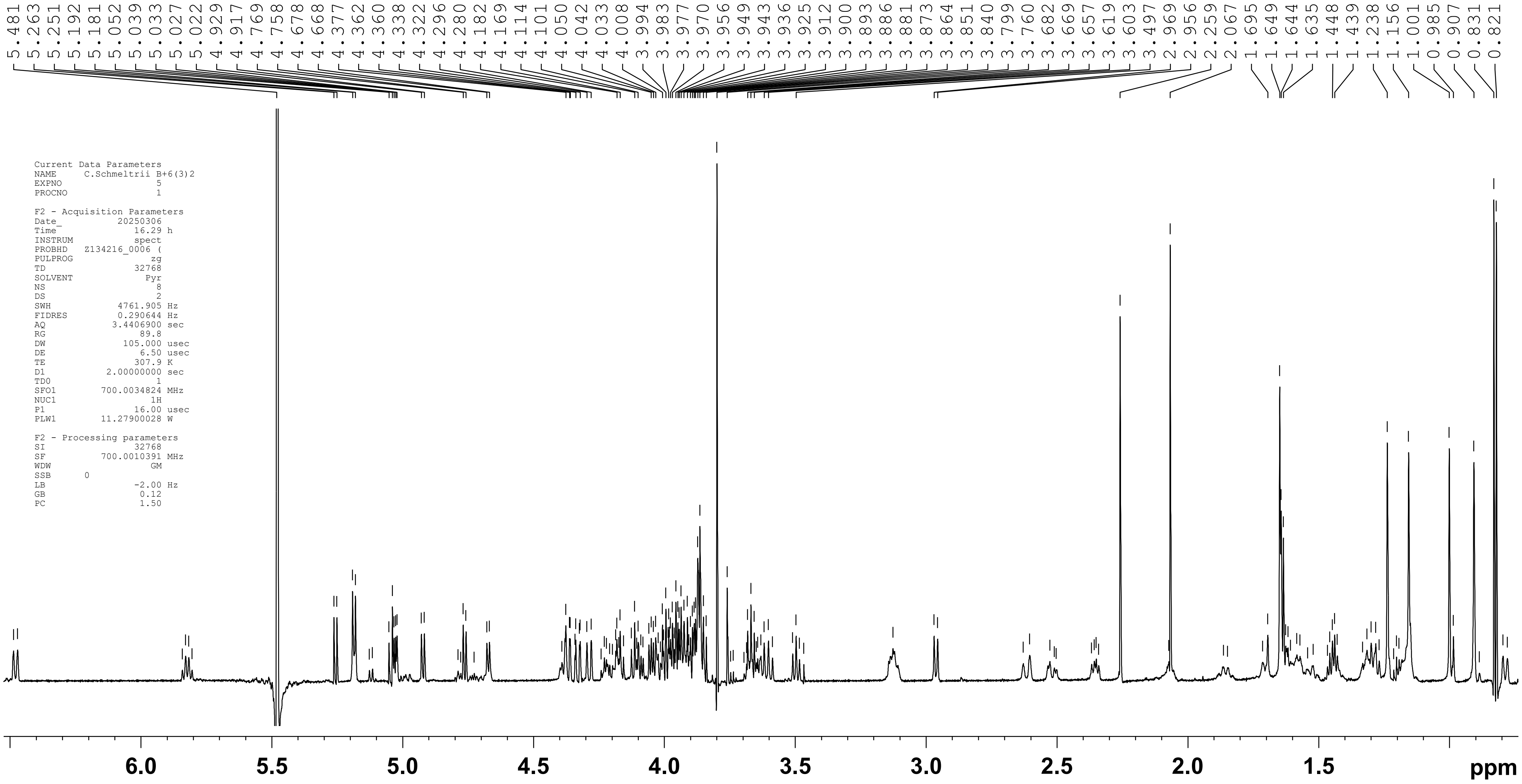

Figure S26. The ^{1}H NMR (700.13 MHz) spectrum of cladoloside T_1 (**4**) in C_5D_5N/D_2O (4/1)

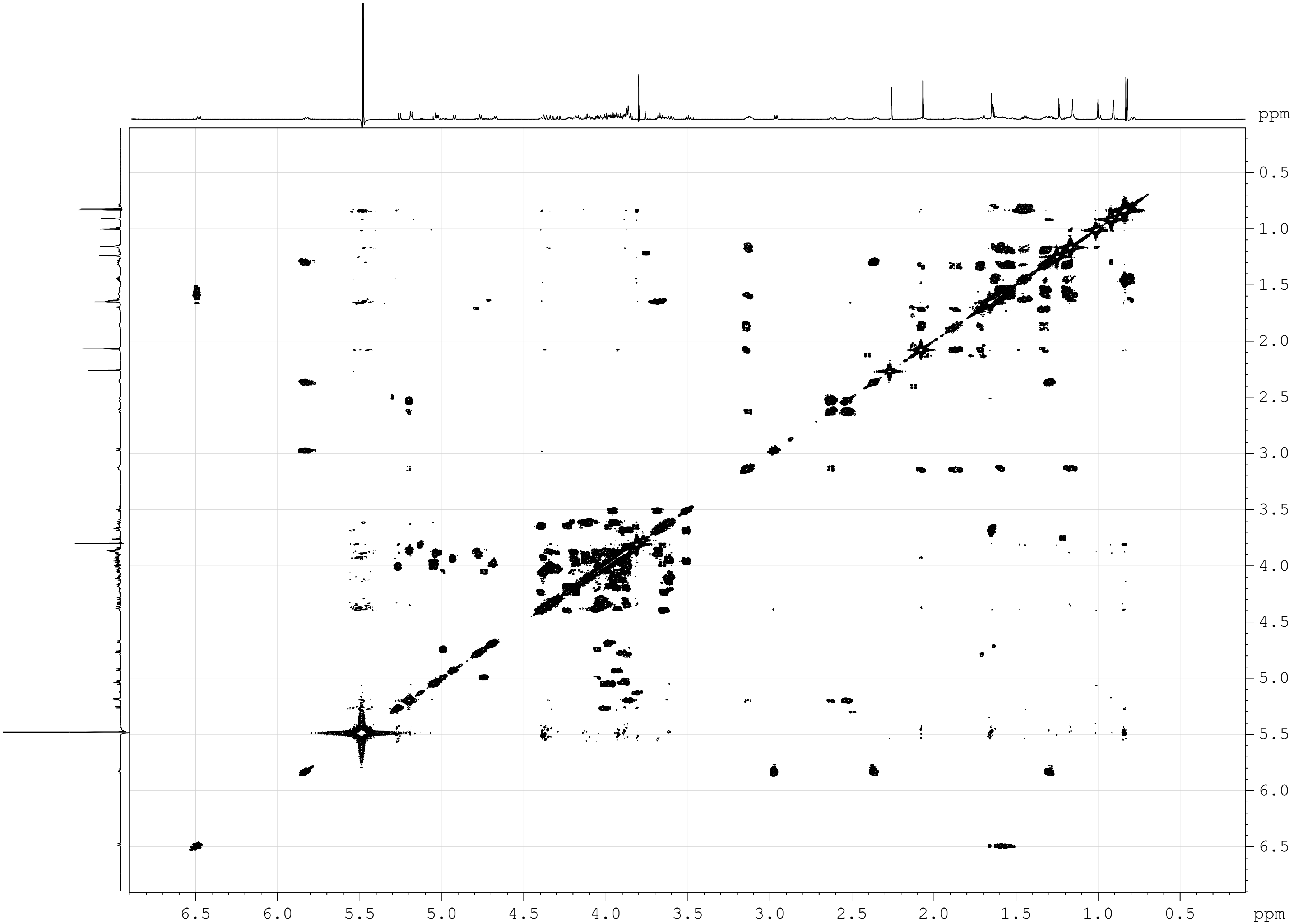

Figure S27. The COSY (700.13 MHz) spectrum of cladoloside T_1 (**4**) in C_5D_5N/D_2O (4/1)

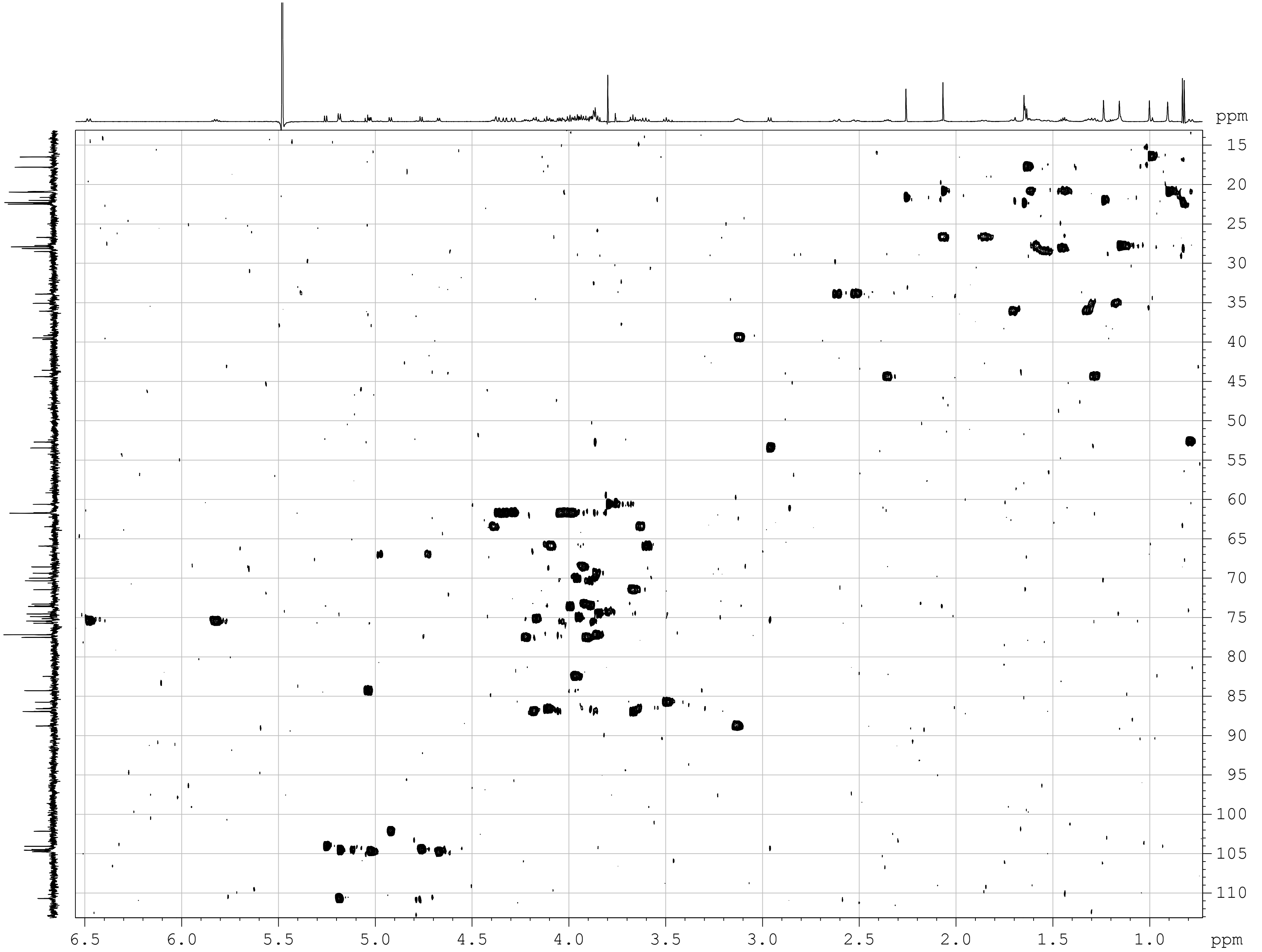

Figure S28. The HSQC (700.13 MHz) spectrum of cladoloside T_1 (**4**) in C_5D_5N/D_2O (4/1)

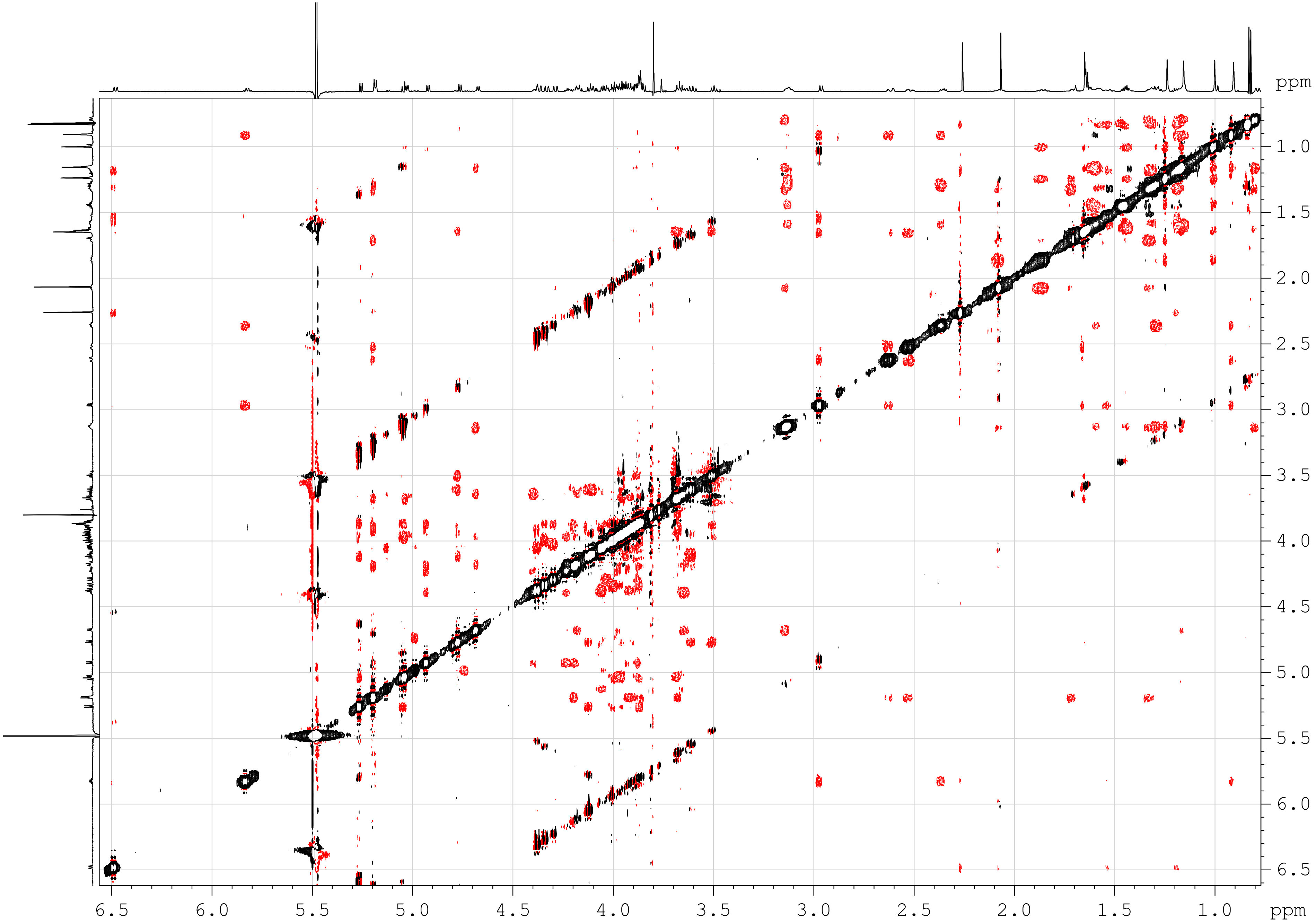

Figure S29. The ROESY (700.13 MHz) spectrum of cladoloside T_1 (**4**) in C_5D_5N/D_2O (4/1)

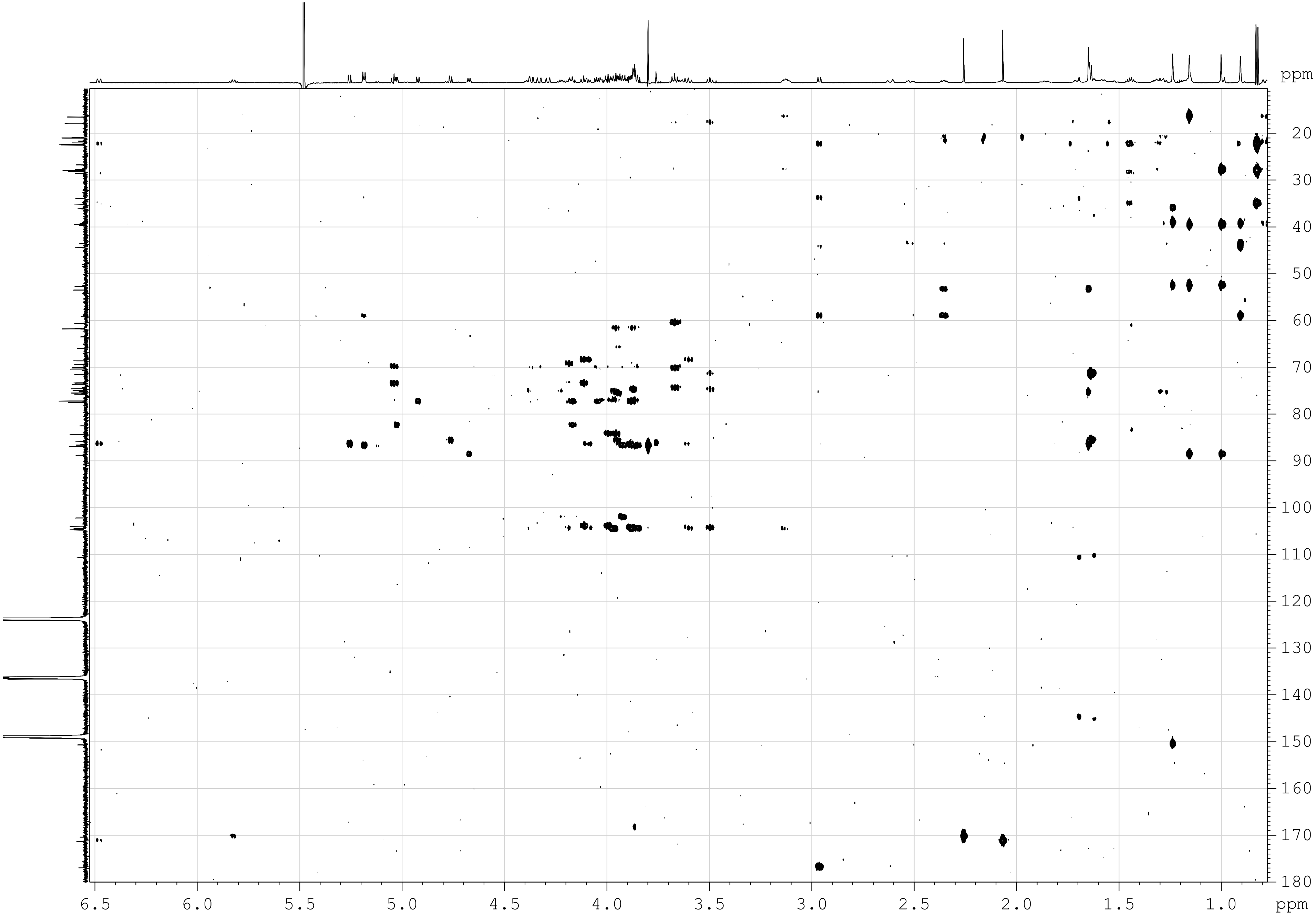

Figure S30. The HMBC (700.13 MHz) spectrum of cladoloside T_1 (**4**) in C_5D_5N/D_2O (4/1)

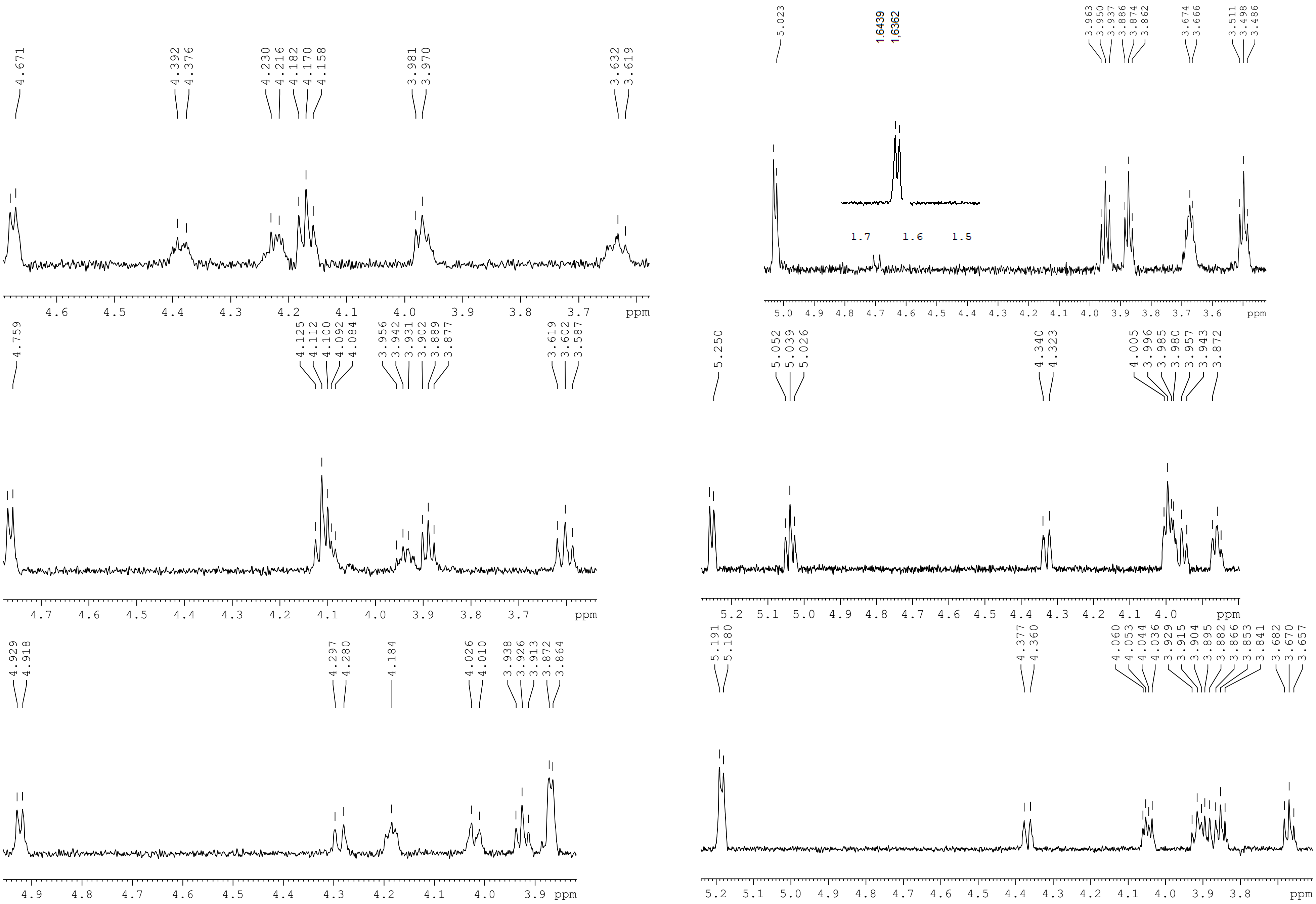

Figure S31. 1 D TOCSY (700.13 MHz) spectra of Xyl1, Qui2, Xyl3, Glc4, Glc5, MeGlc6 of cladoloside T_1 (**4**) in C_5D_5N/D_2O (4/1)

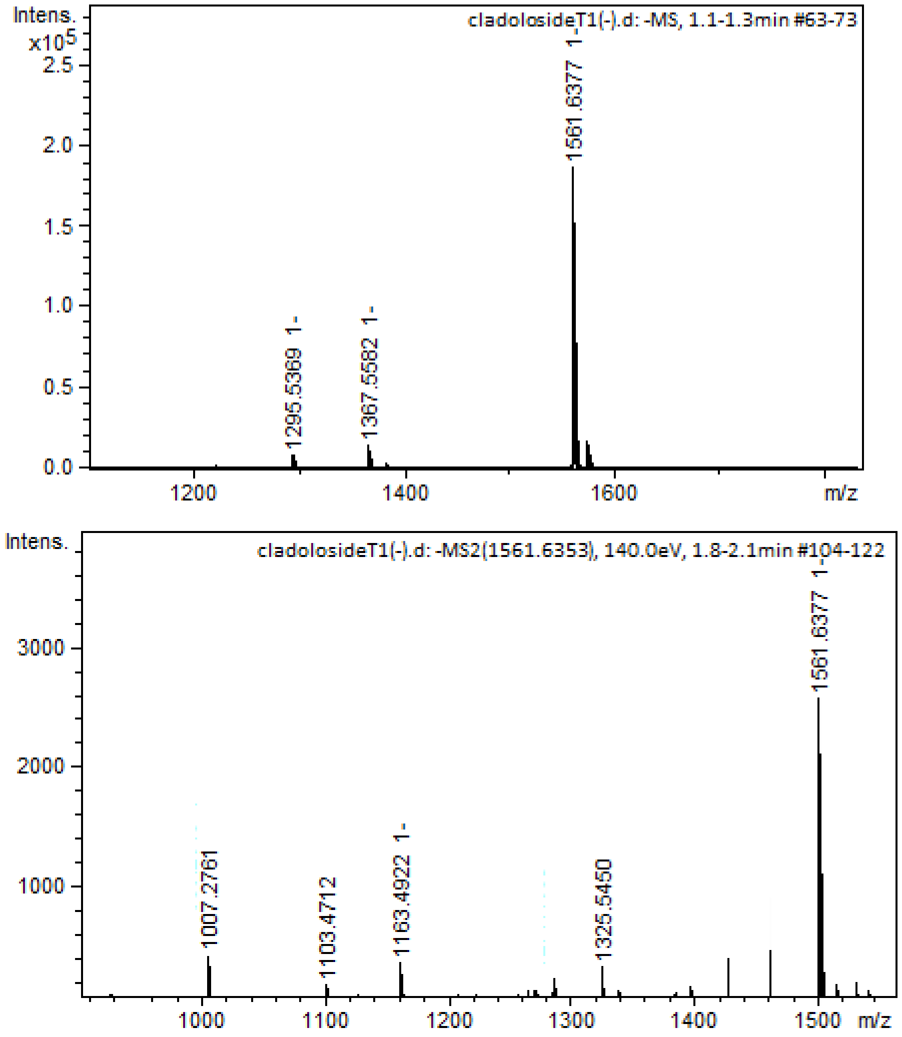

Figure S32. (–)HR-ESI-MS and (–)HR-ESI-MS/MS spectra of cladoloside T1 (**4**)

Table S3. One- and two-dimensional NMR data of the aglycone moiety of cladoloside T_1 (**4**).

Position	δ_C, mult.[a]	δ_H, mult. (*J* in Hz)[b]	HMBC	ROESY
1	36.1, CH_2	1.71, m		H-19
		1.31, m		H-3, H-5
2	26.7,CH_2	2.07, m		
		1.86, m		H-19, H-30
3	88.8, CH	3.13, brd (11.4; 4.2)	C:1 Xyl1	H-1, H-5, H-31, H1-Xyl1
4	39.6, C			
5	52.7, CH	0.79, brd (11.4)		H-3, H-31
6	20.9, CH_2	1.61, m		
		1.44, m	C: 7	
7	27.7, CH_2	1.59, m		
		1.15, m		H-5, H-32
8	39.5, CH	3.11, brd (16.0)		H-15
9	150.7, C			
10	39.2, C			
11	110.7, CH	5.18, m		H-1
12	33.9, CH_2	2.62, brd (16.9)		
		2.52, dd (16.9; 6.0)	C: 17	H-21
13	59.1, C			
14	43.6, C			
15	44.4, CH_2	2.35, dd (12.6; 7.2)	C: 13, 17, 32	H-32
		1.28, brd (12.6)		H-8
16	75.4, CH	5.82, brq (8.9)		H-32
17	53.4, CH	2.96, d (8.9)	C: 12, 13, 18, 21	H-12, H-21, H-32
18	176.9, C			
19	22.0, CH_3	1.24, s	C: 1, 5, 9, 10	H-1, H-30
20	86.5, C			
21	22.4, CH_3	1.65, s	C: 17, 20, 22	
22	75.4, CH	6.48, d (10.5)		
23	28.5, CH_2	1.54, m		
24	35.1, CH_2	1.30, m	C: 22, 26	
		1.17, m		H-22
25	27.9, CH	1.45, m	C: 24	
26	22.5, CH_3	0.83, s	C: 24, 25, 27	
27	22.3, CH_3	0.82, s	C: 24, 25, 26	H-31
30	16.5, CH_3	1.00, s	C: 3, 4, 5, 31	H-2, H-6, H-19, H-30,
31	27.9, CH_3	1.16, s	C: 3, 4, 5, 30	H-1 Xyl1
32	20.9, CH_3	0.91, s	C: 8, 13, 14, 15	H-12, H-15, H-16, H-17, H-24
OAc-16	171.4, C			
	20.9, CH_3	2.07, s		
OAc-22	170.5, C			
	21.6, CH_3	2.25, s		H-22, H-24, H-26, H-27

[a] Recorded at 176.04 MHz in C_5D_5N/D_2O (4/1). [b] Recorded at 700.13 MHz in C_5D_5N/D_2O (4/1).

Table S4. One- and two-dimensional NMR data of the oligosaccharide moiety of cladoloside T_1 (**4**).

Atom	δ_C, mult.[a,b]	δ_H, mult. (*J* in Hz)[c]	HMBC	ROESY
Xyl1 (1→C-3)				
1	104.7, CH	4.67, d (6.9)	C-3	H-3; H-3, 5 Xyl1
2	**82.5,** CH	3.97, t (7.9)	C: 1,3 Xyl1, C: 1 Qui2	H-1 Qui2; H-4 Xyl1
3	75.1, CH	4.17, t (7.9)	C: 2,4 Xyl 1	H-1 Xyl1
4	**77.2,** CH	4.22, m	C: 3 Xyl1, C: 1 Glc5	H-1 Glc5
5	63.5, CH_2	4.39, m	C: 1, 3 Xyl1	
		3.63, m		H-1 Xyl1
Qui2 (1→2Xyl1)				
1	104.7, CH	5.03, d (7.1)	C: 2 Xyl1	H-2 Xyl1, H-3, 5 Qui2
2	75.7, CH	3.87, t (8.5)	C: 1, 3 Qui2	H-4 Qui2
3	74.9, CH	3.95, t (8.5)	C: 2, 4 Qui2	H-1 Qui2
4	**85.7,** CH	3.50, t (8.5)	C: 3, 5 Qui2, 1 Xyl3	H-1 Xyl3, H-2 Qui2
5	71.5, CH	3.67, m		H-1 Qui2
6	17.8, CH_3	1.64, d (6.8)	C: 4, 5 Qui2	
Xyl3 (1→4Qui2)				
1	104.4, CH	4.76, d (7.2)	C: 4 Qui2	H-4 Qui2, H-3, 5 Xyl3
2	73.6, CH	3.89, t (8.8)	C: 1, 3 Xyl3	
3	**86.6,** CH	4.11, t (8.8)	C: 2, 4 Xyl3; 1 Glc4	H-1 Glc4, H-1 Xyl3
4	68.6, CH	3.94, m		
5	65.9, CH_2	4.09, dd (10.4; 5.6)	C: 1, 3, 4 Xyl3	
		3.60, t (10.4)	C: 1, 3, 4 Xyl3	H-1, 3 Xyl3
Glc4 (1→3Xyl3)				
1	104.0, CH	5.26, d (7.9)	C: 3 Xyl3	H-3 Xyl3, H-3, 5 Glc4
2	73.5, CH	4.00, t (8.9)	C: 1, 3 Glc4	
3	*84.3*, CH	5.04, t (8.9)	C: 2, 4 Glc4	H-1, 5 Glc4
4	70.0, CH	3.96, t (8.9)	C: 3, 5 Glc4	H-6 Glc4
5	77.2, CH	3.86, t (8.9)	C: 4 Glc4	H-1, 3 Glc4
6	61.7, CH_2	4.33, dd (2.1; 11.6)	C: 4 Glc4	
		3.99, m	C: 5 Glc4	
Glc5 (1→4Xyl1)				
1	102.2, CH	4.92, d (7.4)	C: 4 Xyl1	H-4 Xyl1, H-3, 5 Glc5
2	73.3, CH	3.92, t (7.9)	C: 1 Glc5	
3	86.9, CH	4.18, t (7.9)	C: 2, 4 Glc5, C: 1 MeGlc6	H-1 MeGlc6; H-1, 5 Glc5
4	69.4, CH	3.87, t (7.9)	C: 3, 5 Glc5	
5	77.5, CH	3.87, t (7.9)	C: 6 Glc5	H-3 Glcl5
6	61.7, CH_2	4.29, brd (11.7)		
		4.02, brd (1173)	C: 5 Glc5	
MeGlc6 (1→3Glc5)				
1	104.5, CH	5.19, d (7.6)	C: 3 Glc5	H-3 Glc5; H-3, 5 MeGlc6
2	74.5, CH	3.85, t (8.9)	C: 1, 3 MeGlc6	
3	86.9, CH	3.67, t (8.9)	C: 2, 4 MeGlc6; OMe	H-1 MeGlc6
4	70.3, CH	3.87, t (8.9)	C: 3, 5 MeGlc6	
5	77.5, CH	3.92, t (8.9)	C: 6 MeGlc6	H-1 MeGlc6
6	61.7, CH_2	4.37, brd (11.9)	C: 4 MeGlc6	H-4 MeGlc6
		4.05, dd (11.9; 6.0)	C: 5 MeGlc6	
OMe	60.6, CH_3	3.80, s	C: 3 MeGlc6	

[a]Recorded at 176.04 MHz in C_5D_5N/D_2O (4/1). [b]Bold = interglycosidic positions. [c]Recorded at 700.13 MHz in C_5D_5N. Multiplicity by 1D TOCSY.

Figure S33. The structure of glycosides, isolated earlier from the sea cucumber *Cladolabes schmeltzii*.

Cladolosides of the groups A

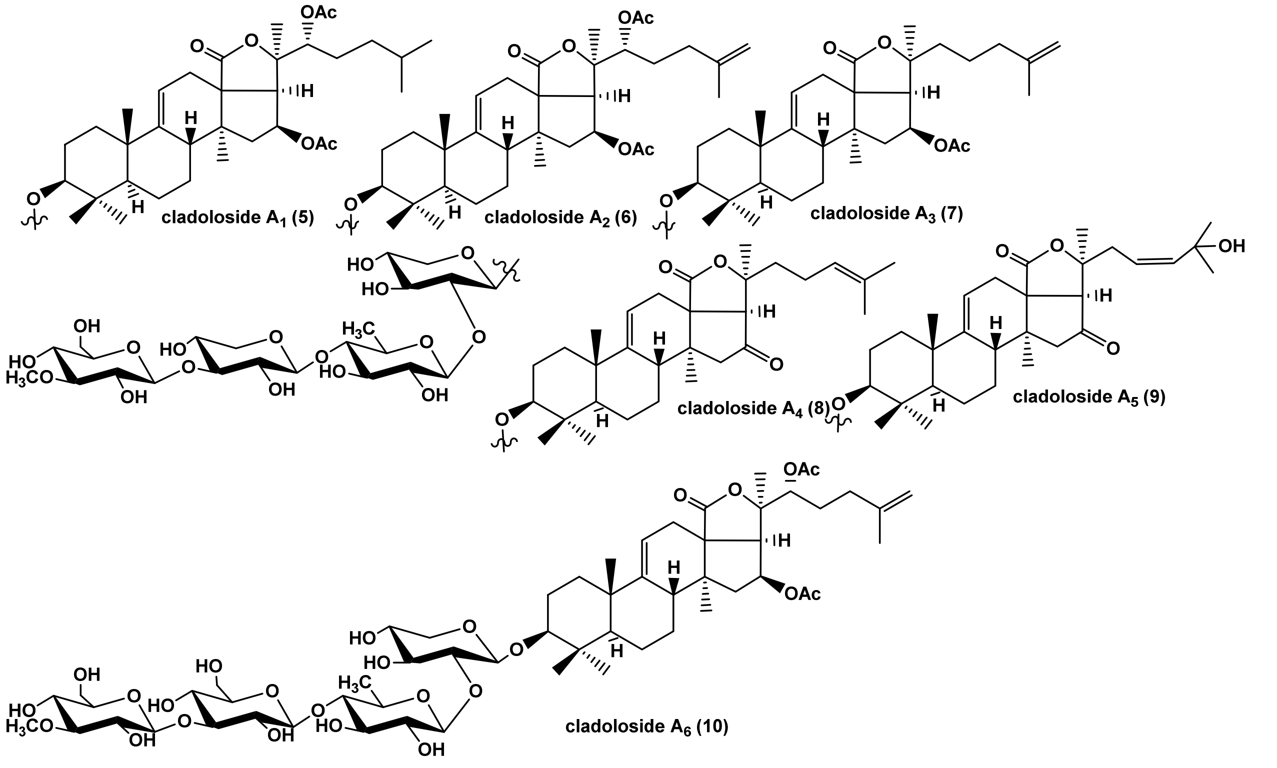

Cladolosides of the group B

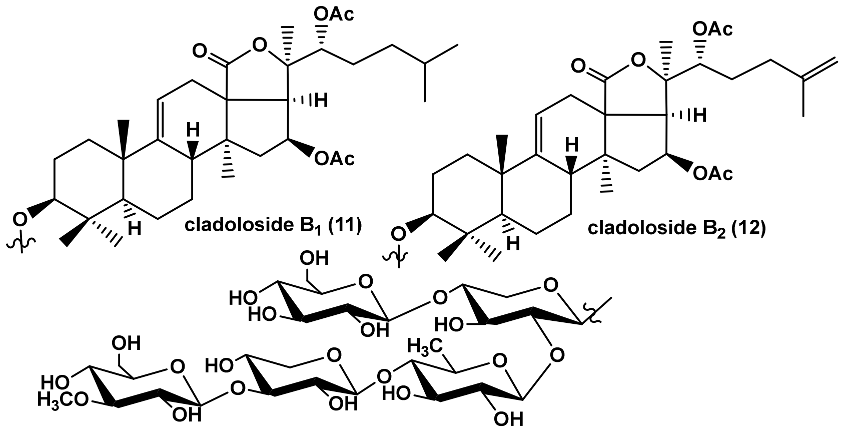

Cladolosides of the group C

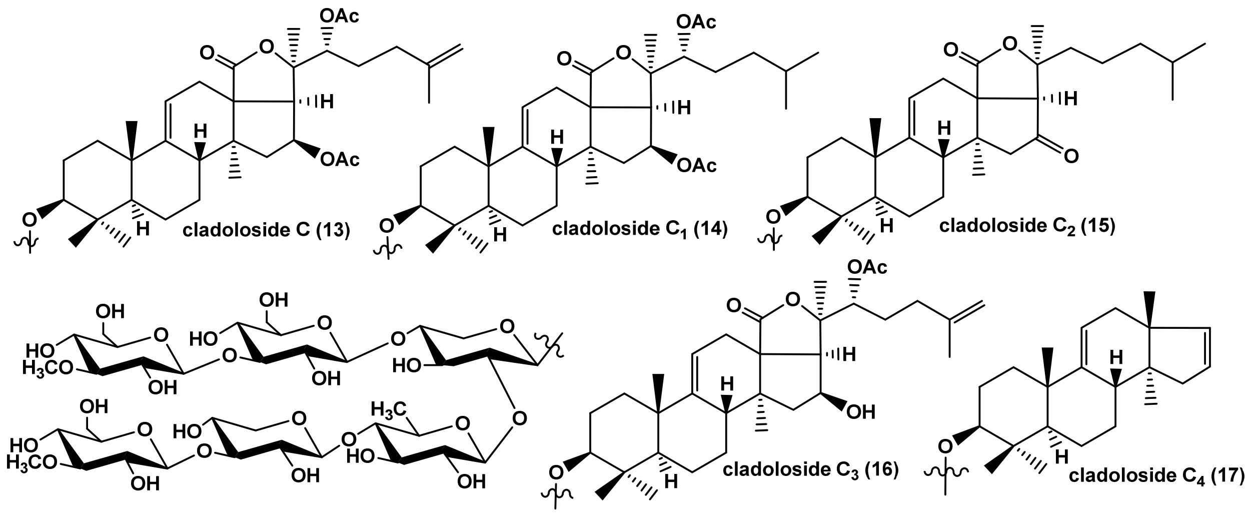

Cladolosides of the group D

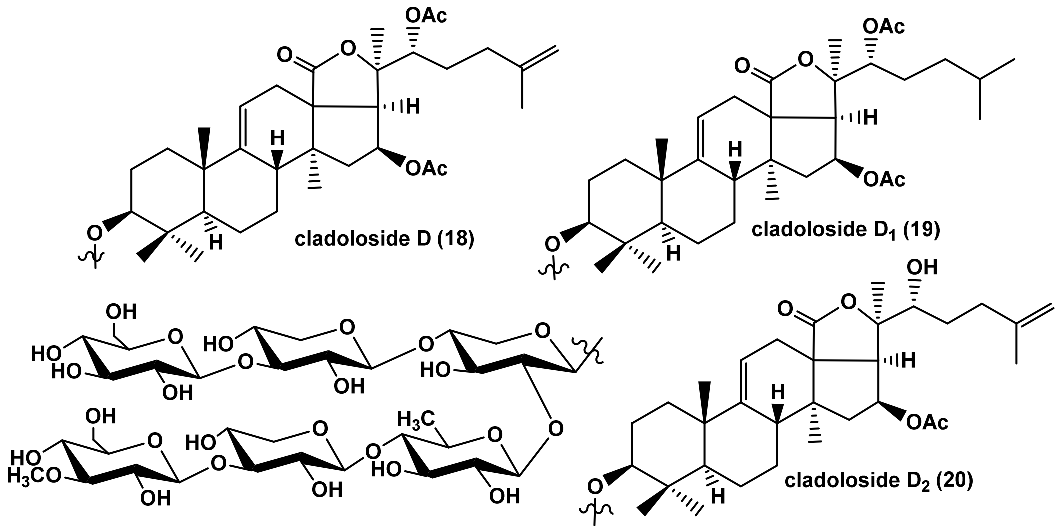

cladoloside D (18)
cladoloside D_1 (19)
cladoloside D_2 (20)

Cladolosides of the group E

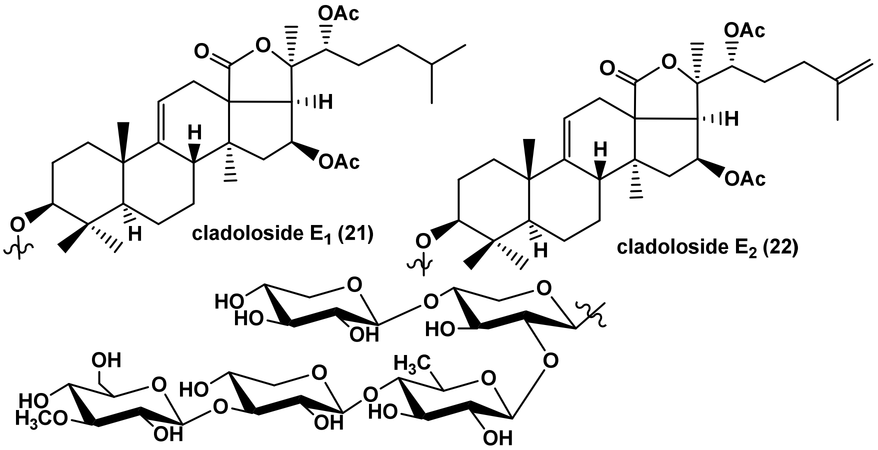

cladoloside E_1 (21)
cladoloside E_2 (22)

Cladolosides of the group F

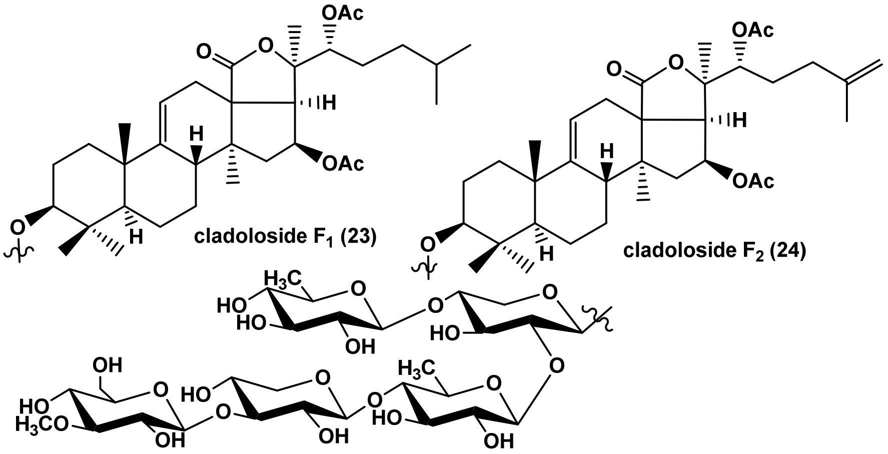

cladoloside F_1 (23)
cladoloside F_2 (24)

Cladoloside G

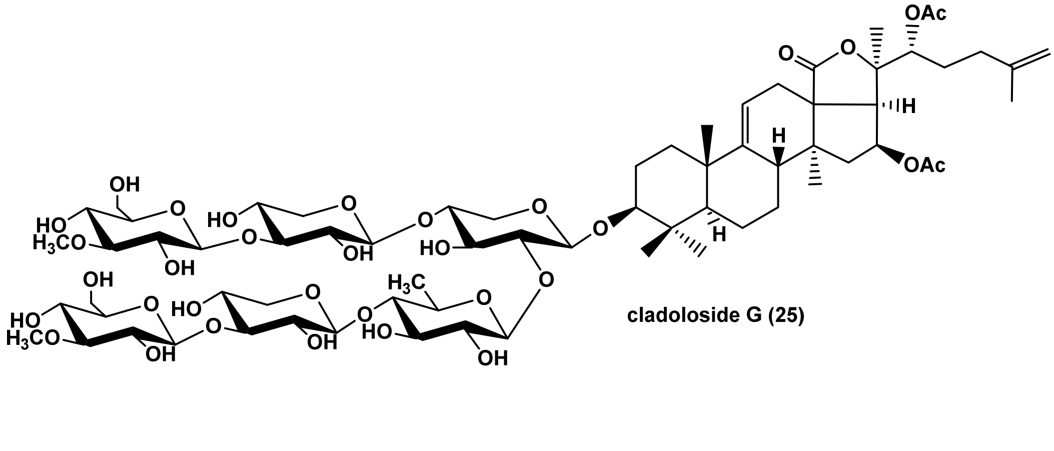

cladoloside G (25)

Cladolosides of the group H

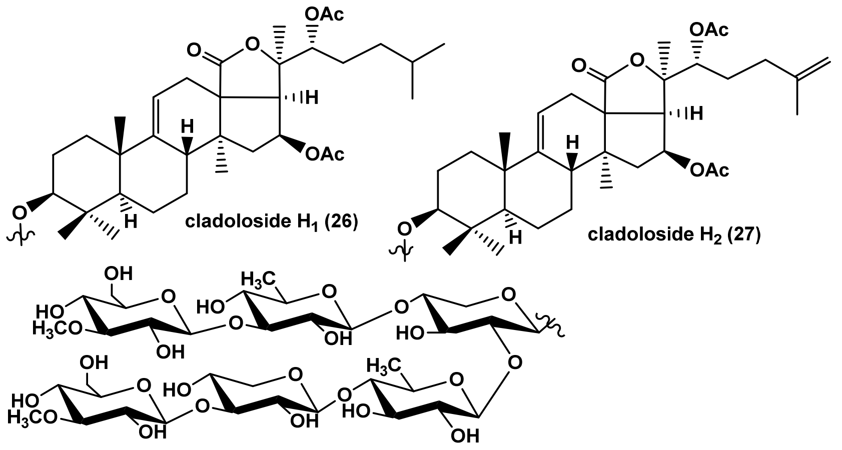

cladoloside H_1 (26)

cladoloside H_2 (27)

Cladolosides of the group I

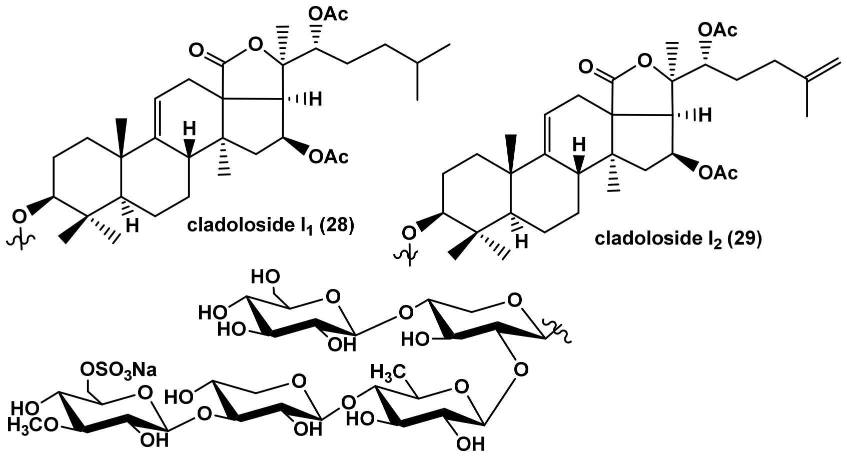

cladoloside I_1 (28)

cladoloside I_2 (29)

Cladoloside J1

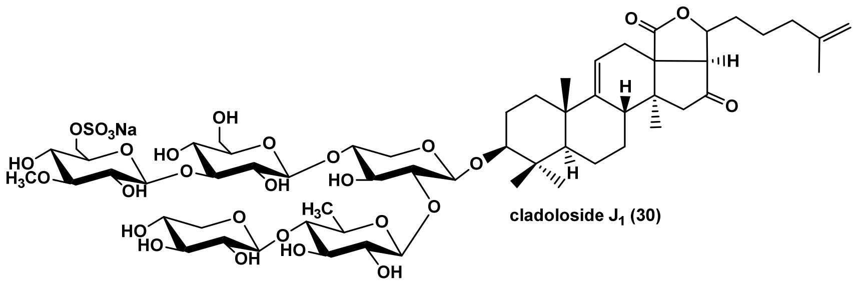

cladoloside J_1 (30)

Cladolosides of the group K

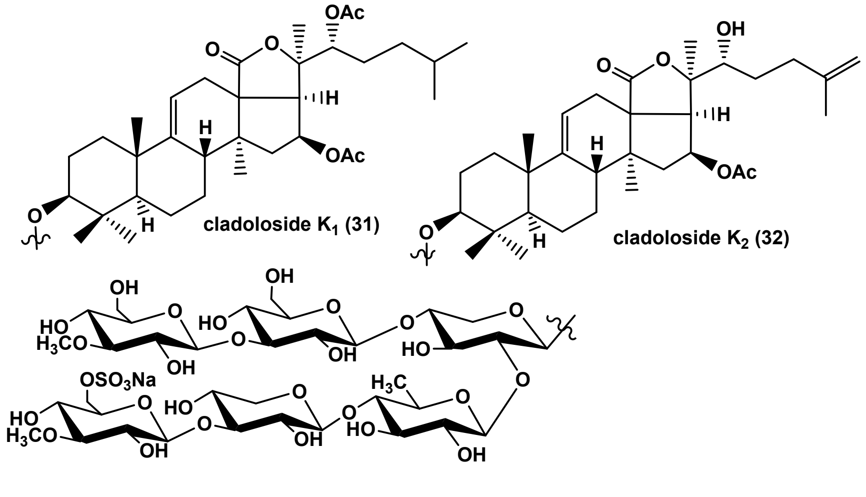

cladoloside K_1 (31)

cladoloside K_2 (32)

Cladoloside L1

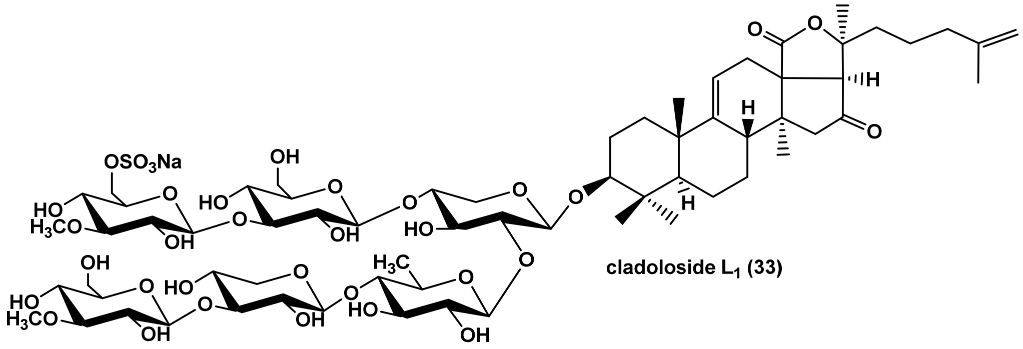

Cladolosides of the group M

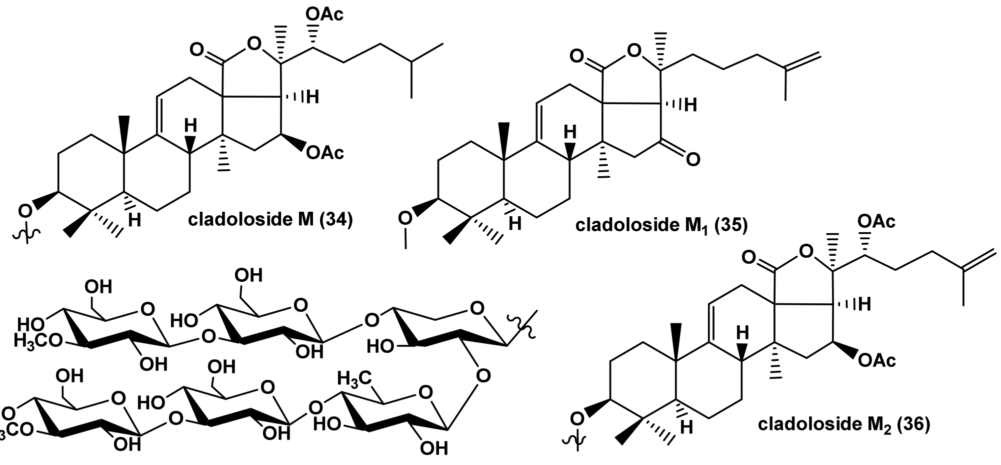

Cladoloside N (37)

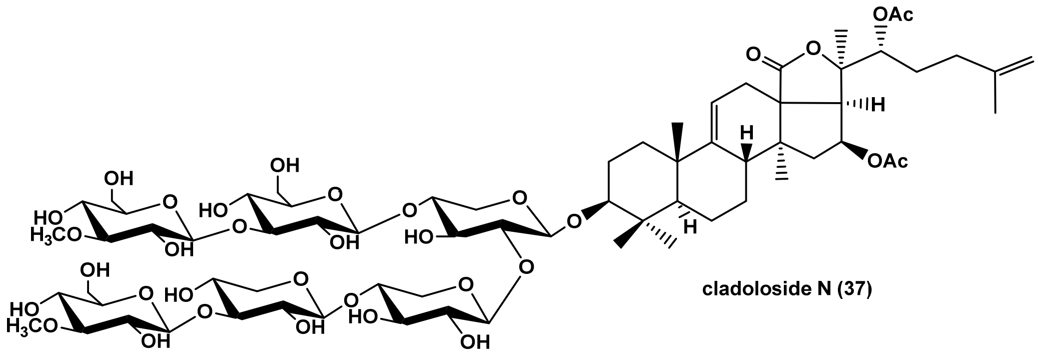

Cladoloside O

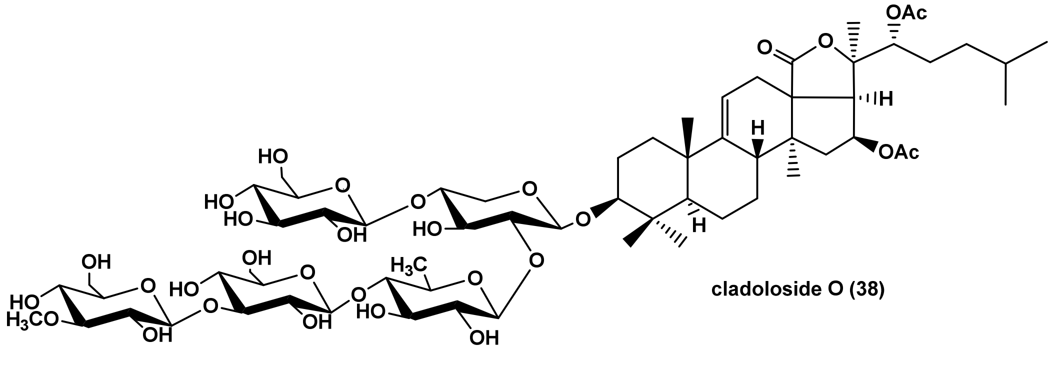

Cladolosides of the group P

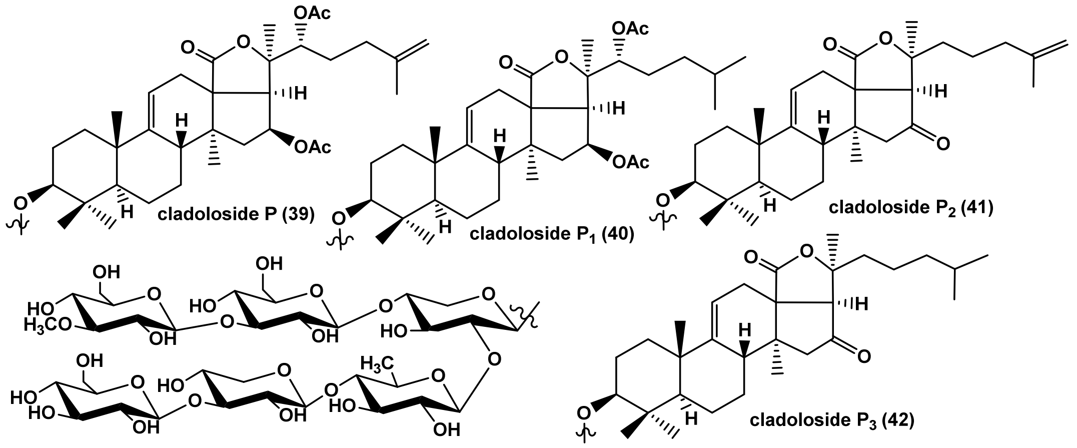

cladoloside P (39) cladoloside P$_1$ (40) cladoloside P$_2$ (41) cladoloside P$_3$ (42)

Cladoloside Q

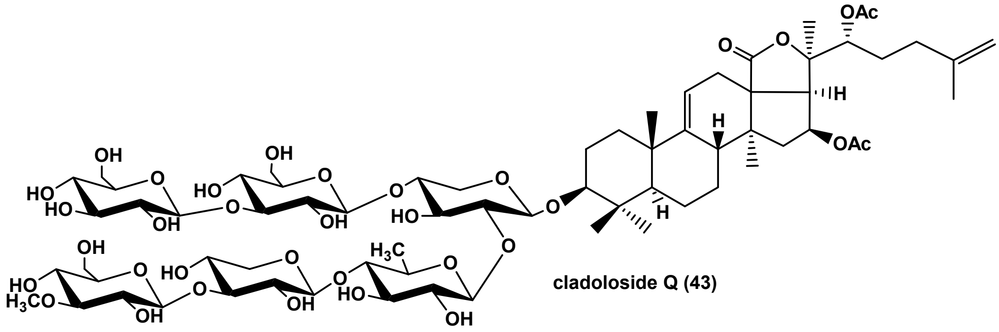

cladoloside Q (43)

Cladoloside R

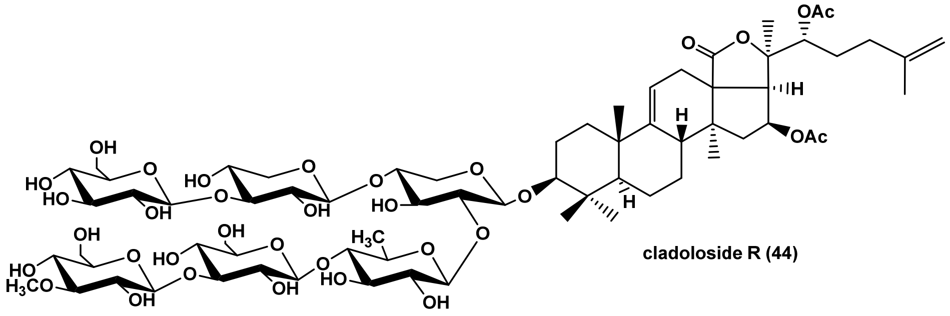

cladoloside R (44)

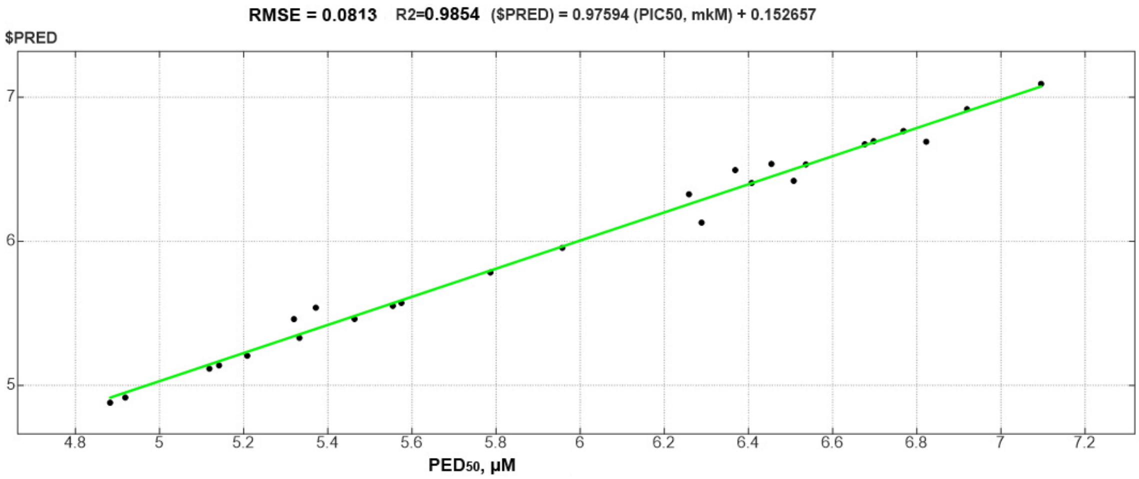

Figure S**34**. The PLS QSAR model correlation plot reflecting the relationship of predicted and experimental cytotoxicity of the glycosides from *C. schmeltzii* against human erythrocytes. The cytotoxic action is expressed as pED_{50}.

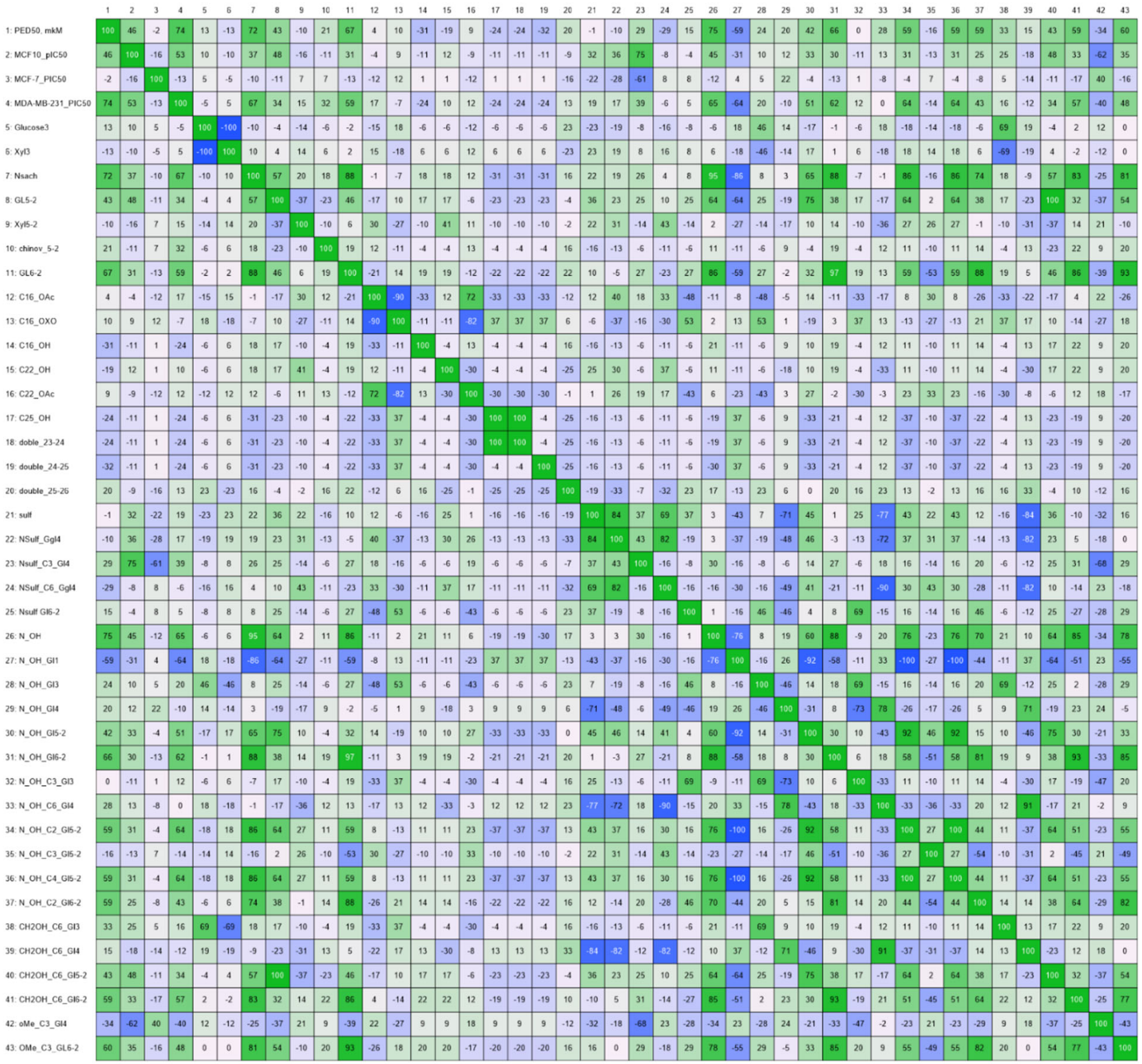

Figure S35. The correlation matrix of descriptors impacts (43 variables) to the hemolytic (pED$_{50}$, µM) and cytotoxic activities (pIC$_{50}$, µM) against MCF-10A, MCF-7 and MDA-MB-231 cells of 26 glycosides constructed with the QuaSAR-Descriptor tool of MOE 2020.0901 CCG software. The following descriptors caused the most pronounced antagonistic effects against MCF-7 and MDA-MB-231 cells: O-acetate (–12 for MCF-7 and +17 to MDA-MB-231) or keto-group (+12 for MCF-7 and –7 to MDA-MB-231) at C-16 of the aglycones, glucose residues in the fifth (–11 for MCF-7 and +34 to MDA-MB-231) and sixth positions (–13 for MCF-7 and +59 to MDA-MB-231), the presence 3-*O*-methyl groups at fourth (+40 for MCF-7 and –40 to MDA-MB-231) and sixth (+35 for MCF-7 and –16 to MDA-MB-231) monosaccharide residues in carbohydrate chains, presence of sulfate groups (–22 for MCF-7 and +19 to MDA-MB-231) and the localization of sulfate group at C-3 of the fourth sugar unit (–61 for MCF-7 cells and +39 for MDA-MB-231).
